# Mitigating cold stress in rice: a study of genotype performance and sowing time

**DOI:** 10.1186/s12870-024-05423-8

**Published:** 2024-07-26

**Authors:** Yasser Z. El-Refaee, Hany S. Gharib, Shimaa A. Badawy, Eman M. Elrefaey, Samira A.F. El-Okkiah, Mohamed K. Okla, María Gabriela Maridueña-Zavala, Hamada AbdElgawad, Amira M. El-Tahan

**Affiliations:** 1 Rice Research Department, Field Crops Research Institute, Agricultural Research Center, Sakha, Kafr El-sheikh, 33717 Egypt; 2 Agronomy Department, Faculty of Agriculture, Kafrelsheikh University, Kafr El-Sheikh, 33516 Egypt; 3 Department of Agriculture Botany, Faculty of Agriculture, Kafrelsheikh University, Kafr El-Sheikh, 33516 Egypt; 4 Botany and Microbiology Department, College of Science, King Saud University, P.O. Box 2455, Riyadh, 11451 Saudi Arabia; 5 Centro de Investigaciones Biotecnológicas del Ecuador (CIBE), Escuela Superior Politécnica del Litoral, ESPOL, Campus Gustavo Galindo, Km. 30.5 Vía Perimetral, Guayaquil, 090902 Ecuador; 6 Department of Biology, Laboratory for Integrated Molecular Plant Physiology Research (IMPRES), University of Antwerp, Antwerp, Belgium; 7 Plant Production Department, Arid Lands Cultivation Research Institute, the City of Scientific Research and Technological Applications, SRTA-City. Borg El Arab, Alexandria, Egypt

**Keywords:** Climate change, Cold tolerance, Rice genotypes, Global food security

## Abstract

Rice (*Oryza sativa* L.) is an essential food for half of the global population and is vital in maintaining global food security. Climate change, increasing population and recent incident of COVID pandemic has generated financial burden and threaten the global food security. Due to theses factors rice cultivation also has to face significant challenges. frequent weather changes pose a considerable challenge to agricultural planning, which was previously relaying on consistent seasonal variations. In this context, rice cultivation is particularly sensitive to cold, where its development and productivity inhibited by low temperatures (< 18 °C). Developing rice varietes with low temprature tolerence and good yield potential is one of the major goals of current breeding efforts of plant scientists. For this purpose, short duration and early rice varieties are most favorable to avoid cold stress and yield more in less number of days. this study was designed to investigate the effect of low temperatures on different rice varieties. the study was designed to identify low temprature tolerent genotypes with early and regular cultivation. For this, thirty-four genotypes were evaluated in two gorwing seasons (2018–2019) with four different sowing times. Statistically sowing time showed significant interaction between all yield contributing parameters. The data indicate that exposure to low temperatures during the reproductive phase prolongs the maturation period of the crop, also length of the panicle and the fertility of the spikelets drops, resulting in a significant decrease in the production of sensitive varieties. Some varieties are more sensitive to cold stress compared to others. In the Egyptian context, Giza176, Sakha104, and Sakha107 are recommended for early cultivation, while the genotypes Giza 179, Sakha101, Sakha104, and GZ 9730-1-1-1-1 are indicated for the normal cultivation period. The Sakha104 variety is particularly notable, as it is recommended for both purposes. In addition, the data obtained in this study provide valuable information for selecting rice varieties suitable for double cropping in the North Delta of Egypt. This study also contributes to the existing literature, providing insights into the resilience of rice cultivation in the face of climate change.

**Supplementary Information:**

The online version contains supplementary material available at 10.1186/s12870-024-05423-8.

## Introduction

Rice, scientifically known as *Oryza sativa* L., is a fundamental dietary component for about 50% of the world’s population. It is crucial in providing essential nutrients for at least one-third of humankind daily [1]. This carbohydrate-rich cereal is also a valuable source of vitamins and minerals such as thiamin, niacin, iron, riboflavin, vitamin D, calcium, and dietary fiber [2]. Additionally, rice provides dietary proteins, though in smaller quantities compared to other grains like soybeans [2, 3].

The COVID-19 pandemic swept across the world in 2020, triggering a global food crisis and warning all governments about the need to ensure food security for their populations [4]. In this context, rice stands out as a food widely consumed in various cultures, with high nutritional value and a capacity for long-term storage [1, 2]. Therefore, rice cultivation holds great dietary and nutritional significance during times of uncertainty. Population growth is also a significant concern for society. According to data from the Food and Agriculture Organization of the United Nations, food production will need to increase by about 50% by 2050 to feed the growing world population [5]. Climate change also poses threat to global food security with frequent fluctuation in temperature and precipitation, makes weather predictability difficult, affects the productive potential of various crops, and adjusts the behavior of multiple pests and diseases [5, 6].

Furthermore, climate change causes difficulties in agricultural planning, which was previously relied on consistent and preditable weather conditions [6]. Rice, originating from tropical and subtropical climates, is a crop sensitive to climate variations. The optimal temperature for its cultivation ranges around 25 to 30 °C, and low temperatures represent one of the principal abiotic stresses affecting the growth and development of rice plants, limiting their production in various countries worldwide [7]. Sensitive to low temperatures at all growth stages, rice shows poor germination, limited growth, and increased plant mortality in cold conditions [8]. Thus, in the last two decades, there has been an increase in research into the molecular and cellular responses of rice plants to this abiotic stress, slightly enhancing the understanding of how cold stress affects the crop [8, 9].

Cold stress in rice leads stunted growth of seedlings, delays flowering, lengthens the growth cycle, reduces tillering, increases plant mortality [10, 11] and causes significant yield loss in rice production [11]. For instance, at the booting stage, low temperatures can also impact panicle branching and pollen fertility [8, 10, 11]. As a result, the number of grains per panicle and seed-setting rate, two crucial components of crop yield, decrease [8, 10]. Low temperatures also induce various physiological changes in reproductive organs, including alterations in another cell walls, affecting cell growth, decreasing viable mature pollen, and increasing sterility [10, 12, 13].

Therefore, one of the objectives of rice breeding programs should be the development of productive varieties with high cold tolerance [14–17]. Increased cold tolerance in crop varieties can lead to more efficient use of the growing season, allowing farmers to plant earlier, harvest later, and potentially fit in an additional crop cycle. This can improve resilience against unpredictable weather patterns associated with shorter growing seasons in colder climates. Thus, the future of rice cultivation worldwide largely depends on the enhancement of cold-tolerant rice varieties and genotypes during the vegetative stage and also on the development of early varieties that can grow well within a short period, even when planted earlier, to avoid adverse weather conditions [14].

In Egypt, the identification of rice genotypes tolerant to cold stress is still in its exploratory stage, and majority of the varieties currently in production are not investigated for their tolerance to cold stress. The general aim of this study is to investigate the effect of exposure of different rice genotypes to low temperatures during reproductive stage of crop development. The main focus was to analyze the impact of cold stress on grain maturation and yield, as well as on panicle length and spikelet fertility, in order to select more cold-tolerant genotypes. To this end, 34 rice genotypes were selected, including a majority of the Egyptian commercial cultivars that are now available, along with many more promising lines. Then the susceptibility and tolerance of 34 rice genotypes to cold stress were investigated to identify suitable rice genotypes for early and regular cultivation in Egypt to avoid cold stress, with the intent of improving grain yield under cold conditions. Consequentially, this will contribute in the development of cold-tolerant rice varieties suitable for cultivation in the Northern Delta region of that country.

## Materials and methods

### Study location and air temperature in the experiment field

**Fig. 1:**
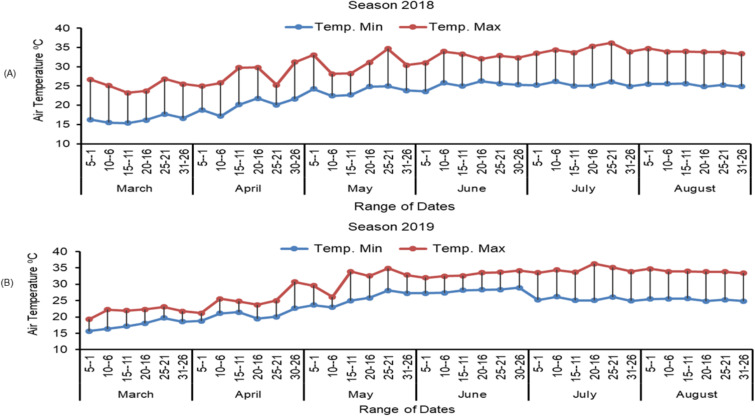
Air temperatures during the trial growing seasons in 2018 (**A**) and 2019 (**B**)

Experiments were conducted at the experimental farm of the Rice Research Department at Sakha Agricultural Research Station, Kafer El-Sheikh Governorate, Egypt. The experiments were performed during the two growing seasons (2018 and 2019). The coordinates of the field experiment site are as follows: longitude (south, 30° 56’ 47”; latitude (east, 31° 21’ 25”) (source:http://www.maphill.com/egypt/lower-egypt/kafr-el-sheikh/location-maps/shaded-relief-map/). During the trial, air temperatures were recorded with digital thermometers (TGP-4520, Gemini Data Loggers, 2011, Chichester, England) and a nearby weather station. Digital thermometers positioned in two distinct blocks tp measure the daily air temperatures (Fig. 1). Throughout the trials, the temperature ranged from a low of 15 °C in March to a high of 40 °C in July (Fig. 1). Low air temperatures were recorded early in the growing season (March to April), while the maximum temperatures were recorded in June and August. The relative humidity was high in the morning, ranging from 70 to 90%, and low at midday, fluctuating between 40% and 60%.

### Soil and irrigation water properties

Used soil was 55% clay, 32.4% silt, and 12.6 sand. Furthermore, the chemical properties of the soil and irrigation water for the experimental site were recorded in Table 1.

**Table 1:** Chemical properties of soil and irrigation water for the experimental site

Soil and water		EC (ds/m)	pH	Anions (mq/L)				Cations (mq/L)				O.M. %	SAR
				Co3	HCo3	Cl-	So4-	Ca++	Mg++	Na+	K+		
Soil	2018	3.35	8.1	--	5.55	14.81	65.8	16.94	4.64	1.82	16.3	1.37	7.43
	2019	3.37	8.4	--	5.58	15.2	66.5	16.55	5.07	1.67	16.2	1.44	7.35
Irrigation water	2018	0.56		2.11	18.15	2.14	7.61	4.65	4.67	4.82	0.24		2,25
	2019	0.64		2.07	17.67	2.23	7.73	4.76	4.94	5.13	0.25		2.41

EC, electrical conductivity; OM, organic matter; SAR, sodium adsorption ratio

### Plant materials, field procedures, and experimental design

In the present research, thirty-four rice genotypes were selected, including a majority of the Egyptian commercial cultivars that are now available, along with many more promising lines. These included genotypes of temperate *japonica*, *indica*, and *indica* x *japonica* origin, as well as an exotic indica genotype. The seeds were procured from the Egyptian Rice Germplasm Unit (ERGU), the Rice Research Department within the Field Crops Research Institute, the Agricultural Research Centre in Sakha, Egypt. The nomenclature and provenance of the genotypes under investigation were reported (Table 2). After thirty days, seedlings representing each genotype were meticulously replanted into three distinct rows inside the experimental field. Each row had a length of 5.0 m, with a gap of 20 centimeters between rows and hills. During all growth seasons, the experimental populations were planted on four distinct sowing dates including March 1st (SD1), March 20th (SD2), April 10th (SD3), and May 1st (SD4). SD1 and SD2 were established in order to synchronize the vegetative and reproductive phases of rice cultivation, therefore exposing them to low temperatures (cold stress). SD3 exhibited an intermediate position relative to both the conventional and early sowing dates. SD4 is a typical planting date which was characterized by the rice crop’s ability to develop from seedling to harvest without being subjected to cold stress. A randomized full-block design was used, consisting of three repetitions. Each replication included two control genotypes: Giza 177 (tolerant japonica genotype) and IR 83,106-B-B-2 (sensitive indicia genotype). Conventional agricultural methods, including plowing the land, irrigation till flooding, adding the fertilizers, management the weed and monitoring the pest, were used for rice cultivation in both growing seasons. Data pertaining to the genotypes of 10 guarded plants were selected in a random manner from each plot. These data were duly documented by observations and measurements.

**Table 2:** List of thirty-four rice genotypes used in this study

No.	Genotypes	Type	source	No.	Genotypes	Type	source
1	Giza 171	J	ERGU	18	MILYANG 240	I	IRRI
2	Giza 172	J	ERGU	19	Korea 14	J	IRRI
3	Giza 176	J	ERGU	20	IR 83,106-B-B-2	I	IRRI
4	Giza 177	J	ERGU	21	SKC 2015-1	J	ERGU
5	Giza 178	I/J	ERGU	22	SKC 2015-2	J	ERGU
6	Giza 179	I/J	ERGU	23	GZ 9730-1-1-1-1	J	ERGU
7	Sakha 101	J	ERGU	24	GZ 9730-1-1-3-2	J	ERGU
8	Sakha 102	J	ERGU	25	GZ 9626-2-1-3-2	J	ERGU
9	Sakha 104	J	ERGU	26	GZ 6296-12-1-2-1	I	ERGU
10	Sakha 105	J	ERGU	27	IET 1444	I	IRRI
11	Sakha 106	J	ERGU	28	GZ 1368-S-5-4	I	ERGU
12	Sakha 107	J	ERGU	29	GZ 6903-1-2-2-1	J	ERGU
13	Reiho	J	ERGU	30	CIASEM	I	IRRI
14	HR 20654-54-3-5	J	IRRI	31	Sanakevelle	I	IRRI
15	IR 68,333-R-R-B-19	I	IRRI	32	Carola	J	IRRI
16	IR 11 K 305 A	I	IRRI	33	I Geo Tze	J	IRRI
17	IR 12 K 269	I	IRRI	34	WOMBAT	J	IRRI

J = japonica type, I = indica type, I/J = indica/japonica type, ERGU = Egyptian Rice Germplasm Unit; IRRI = International Rice Research Institute

### Traits measurements

The agro-morphological features were assessed in accordance with the Standard Evaluation System of the International Rice Research Institute (IRRI) [18]. The variables that were measured in this study included heading date, plant height (cm), panicle length (cm), number of productive tillers per plant, fertility percentage, harvest index, grain yield per plant (g), and biomass (g). The heading date was determined as the number of days from sowing until 50% of all panicles in each plot had emerged. Plant height was assessed at maturity by measuring from the soil surface to the top of the primary panicle, excluding awns. Panicle length was defined as the distance from the base to the tip of the main panicle at full heading, excluding any awns. The quantification of productive tillers per plant and the overall count of tillers per hill were conducted to reach the full ripeness of all panicles. To determine the fertility percentage, the filled grains from the primary panicle were isolated and enumerated. The fertility percentage was then computed using the following formula:

$${\rm{Fertility}}\,{\rm{\% = }}\left( {{\matrix{{\rm{Number}}\,{\rm{of}}\,{\rm{filled}}\, \hfill \cr {\rm{grains}}\,{\rm{per}}\,{\rm{panicle}} \hfill \cr} \over \matrix{{\rm{Number}}\,{\rm{of}}\,{\rm{total}}\, \hfill \cr {\rm{spikelets}}\,{\rm{per}}\,{\rm{panicle}} \hfill \cr} }} \right){\rm{ \times 100}}$$

The harvest index (HI) was calculated as follows:

$${\rm{HI}}\,{\rm{\% = }}\left( {{{{\rm{Grain}}\,{\rm{yield}}} \over \matrix{{\rm{Biomass}}\,{\rm{yield}}\,{\rm{(straw}}\,{\rm{weight}}\, \hfill \cr + \,{\rm{grain}}\,{\rm{weight)}} \hfill \cr} }} \right) \times 100$$

### Statistical analysis of data

The statistical technique known as analysis of variance (ANOVA) was used to examine and compare the various assessed features. Each planting date and its repetitions are considered an independent experiment. When the assumption of error homogeneity could not be discarded, a combined analysis was conducted over the four sowing dates in the two seasons [19]. The genotypes’ means were statistically compared using the Least Significant Difference (LSD) test at a significance threshold of 0.05 [20]. The statistical analysis was conducted using the statistical functions provided by both Microsoft EXCEL (2016) and GenStat 18 [21]. The R statistical program (version 4.1.1) was used to implement the heatmap, hierarchical clustering, and principal component analyses.

## Results

### Assessment of interaction between sowing dates and the genotypes for studied agro-morphological traits

The analysis of variance for the studied characters across the two growing seasons, sowing dates, and studied rice genotypes is presented in Table 3. The mean squares due to sowing dates and genotypes were highly significant for all the studied characters. The four sowing dates showed sufficient genetic variability among the studied genotypes. The growing seasons did not significantly impact most studied traits, except for biomass. However, significant interactions between seasons, sowing dates, and genotypes were observed for panicle length, number of productive tillers per plant, fertility percentage, harvest index, and biomass.

**Table 3:** Analysis of variance for the agro-morphological characters across the seasons, sowing dates, and studied rice genotypes

SOV	df	Heading date		Plant height		Panicle length		Productive tillers	
		MS	*P*-value	MS	*P*-value	MS	*P*-value	MS	*P*-value
Seasons (S)	1	0.197^ns^	0.777	0.0542^ns^	0.842	0.001^ns^	0.977	0.603^ns^	0.590
Sowing dates (SD)	3	13,064**	0.000	12667.18**	0.000	483.904**	0.000	2396.71**	0.000
S*SD	3	26.660**	0.000	14.212**	0.000	0.258**	0.000	0.428**	0.000
Rep/SD/S = (Ea)	16	2.370		1.324		1.197		1.992	
Genotypes (G)	33	1995**	0.000	2467.824**	0.000	42.211**	0.000	141.23**	0.000
G*S	33	2.213**	0.001	2.976**	0.000	0.128^ns^	1.000	0.351^ns^	1.000
G*SD	99	46.822**	0.000	34.823**	0.000	2.7137**	0.000	6.922**	0.000
G*S*SD	99	3.299**	0.000	1.561**	0.000	0.135**	0.000	0.374**	0.000
Pooled Error = (Eb)	528	1.126		1.386		0.819		1.119	
Total	815								
**SOV**	**df**	**Fertility %**		**Harvest Index**		**Grain Yield /plant**		**Biomass**	
		**MS**	***P*** **-value**	**MS**	***P*** **-value**	**MS**	***P*** **-value**	**MS**	***P*** **-value**
Seasons (S)	1	24.444^ns^	0.066	13.07^ns^	0.065	5.151**	0.000	76.517*	0.011
Sowing dates (SD)	3	7450.22**	0.000	9819.99**	0.000	2738.60**	0.000	24922.81**	0.000
S*SD	3	4.746**	0.000	7.708**	0.000	7.093**	0.000	1.178**	0.000
Rep/SD/S = (Ea)	16	6.293		0.674		1.314		9.153	
Genotypes (G)	33	78.209**	0.000	282.835**	0.000	44.602**	0.000	1080.14**	0.000
G*S	33	2.359^ns^	0.383	2.489^ns^	0.958	0.887**	0.000	9.428^ns^	0.318
G*SD	99	14.04**	0.000	24.051**	0.000	6.734**	0.000	65.613**	0.000
G*S*SD	99	1.377**	0.000	0.824**	0.000	0.781**	0.000	6.284**	0.000
Pooled Error = (Eb)	528	2.231		0.592		1.451		8.532	
Total	815								

### The effect of low temperatures on rice morphological traits

#### Heading date

**Fig. 2:**
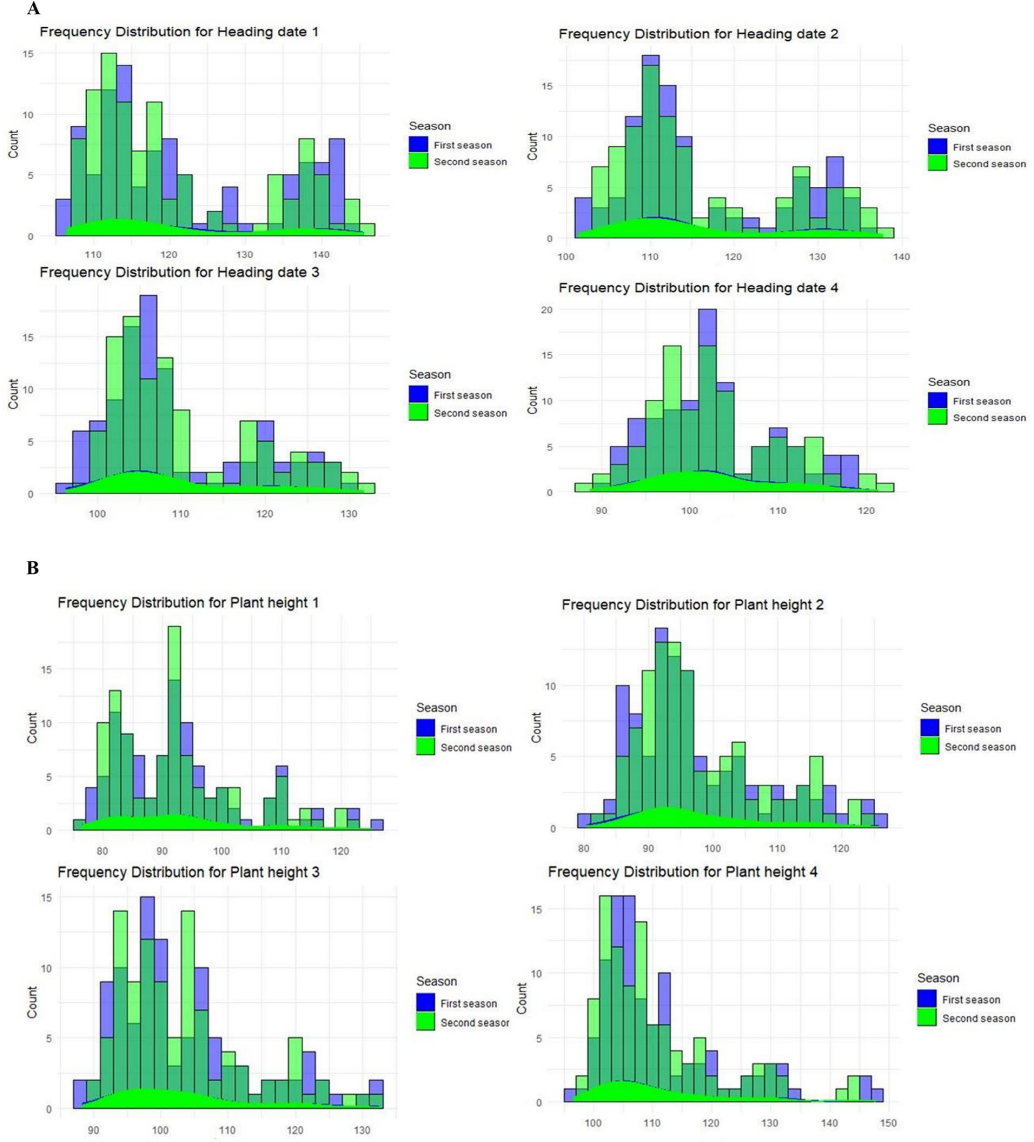
(**A**) Frequency distribution for a set of the studied genotypes for heading dates (days) for the four different sowing dates (І, ІІ, ІІІ, and ІV) over the two growing seasons. (**B**) Frequency distribution for a set of the studied genotypes for plant height (cm) for the four different sowing dates (І, ІІ, ІІІ, and ІV) over the two growing seasons

Low-temperature stress delayed the heading date by lengthening the duration of flowering. On the first sowing date (SD1-SD4), the heading date for the variety Giza 171 was recorded at 144.12 days. In comparison, the heading dates for the subsequent three sowing dates were 135.27, 129.70, and 119.49 days, respectively (Table S1 and Table S2). More than 10% of the genotypes matured after 140 days from sowing on SD1 (Fig. 2AI), whereas approximately 20% matured after 130 days from sowing on SD2 (Fig. 2AII). About 10% of genotypes matured over 120 days after sowing on SD3 under light cold stress (Fig. 2AIII), while less than 10% matured in less than 120 days after sowing on SD4 without cold stress (Fig. 2AIV). The genotypes IR 11K305A, SKC 2015-1, Carola, HR20654-54-3-5, and IR 68,333-R-R-B-19 had a heading date of fewer than 110 days for all four SDs (Table S1 and Table S2). For Giza 177, maturity occurred approximately 110 days from sowing on SD1 and SD2. Giza 171, IR 83,106-B-B-2, CIASEM, Giza 172, and Giza 176 showed heading dates exceeding 130 days from sowing on SD1 and SD2 (Table S1 and Table S2).

#### Plant height

During SD1 and SD2, when low-temperature stress coincided with the vegetative stage, many genotypes, including some sensitive ones, exhibited stunted growth. About 30% of the entries had less than 90 cm of plant height, however, the low-temperature effect was more severe on SD1, exhibiting 30% of the genotypes had less than 90 cm of plant height (Fig. 2BI). While on SD2, about 30% had less than 90 cm of plant height (Fig. 2BII, III, IV), and about 30% of the genotypes (subjected to light cold stress) had less than 100 cm on SD3. The Sanakevelle and WOMBAT genotypes had more than 115 cm of plant height for SDs (Table S1 and Table S2). When the cold stress occurred for the first sowing date, eleven genotypes showed a plant stature of at least 85 cm, including the sensitive check variety (IR 83,106-B-B-2) (Table S1 and Table S2).

#### Panicle length

**Fig. 3:**
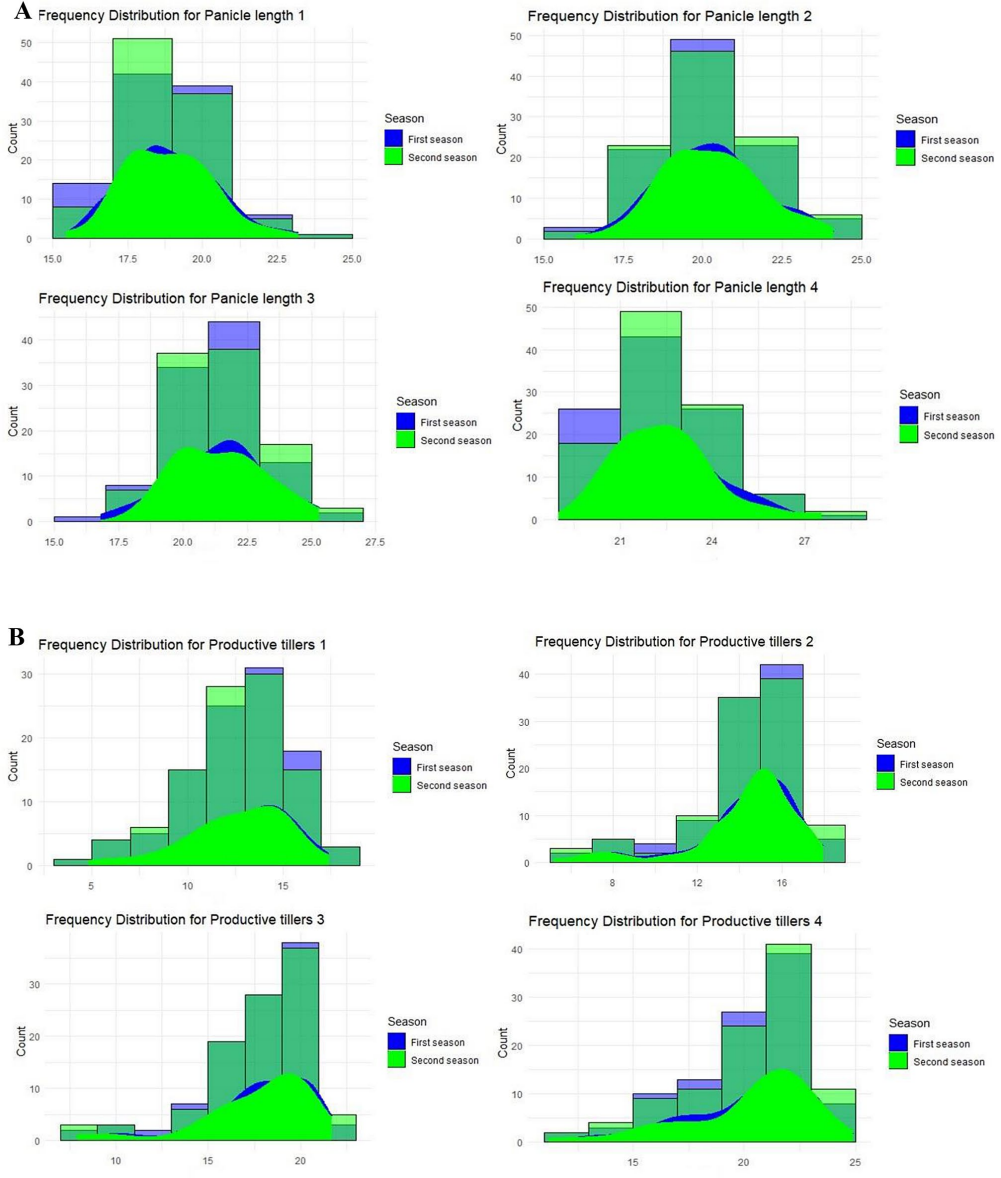
(**A**) Frequency distribution for a set of the studied genotypes for panicle length (cm) for the four different sowing dates (І, ІІ, ІІІ, and ІV) over the two growing seasons. (**B**) Frequency distribution for a set of the studied genotypes for productive tillers for the four different sowing dates (І, ІІ, ІІІ, and ІV) over the two growing seasons

More than 20% of the studied genotypes had panicles less than 18 cm on SD1 (Fig. 3AI). Compared with SD3 and SD4, the panicle length was shorter when cold stress occurred during the reproductive stage (SD2), with more than 20% of the genotypes exhibiting panicles with less than 20 cm (Fig. 3AII, III, IV). The Sanakevelle and CIASEM genotypes had more than 21 cm of panicle length for SDs (Table S2). According to the maximum difference between the lowest and highest panicle length across the values for SDs, Sakha 102, SKC2015-2, Sakha 105, and IR 83,106-B-B-2 were affected by cold stress (the sensitive check) (Table S3 and Table S4).

#### Productive tillers

About 20% of the studied genotypes had less than 12 productive tillers/plants on SD1(Fig. 3BI). For SD2, the number of productive tillers/plants decreased compared with SD3 and SD4, with more than 20% of the studied genotypes having fertile tillers of less than 14 tillers/plants (Fig. 3BII). More than 20% of the genotypes assessed had more than 18 productive tillers/plants for SD3 and SD4, which were not subjected to cold stress (Fig. 3BIII and IV). The GZ6296-12-1-2-1 and CIASEM genotypes had over 15 productive tillers/plants for all SDs (Table S3 and Table S4). The genotypes most impacted by cold stress, ranked by the maximum difference between the lowest and highest values of productive tillers between the second and third sowing dates, were Carola, GZ 1368-S-5-4, MILYANG 240, IR 11 K 305 A, IR 68,333-R-R-B-19, and IR 83,106-B-B-2 (Table S3 and Table S4). The genotypes showing the most insufficient fertile tillers differences between SD2 and SD3 were Sanakevelle, followed by Giza 177 (the tolerant check) and Sakha 102 (Table S3 and Table S4).

#### Spikelet fertility

**Fig. 4:**
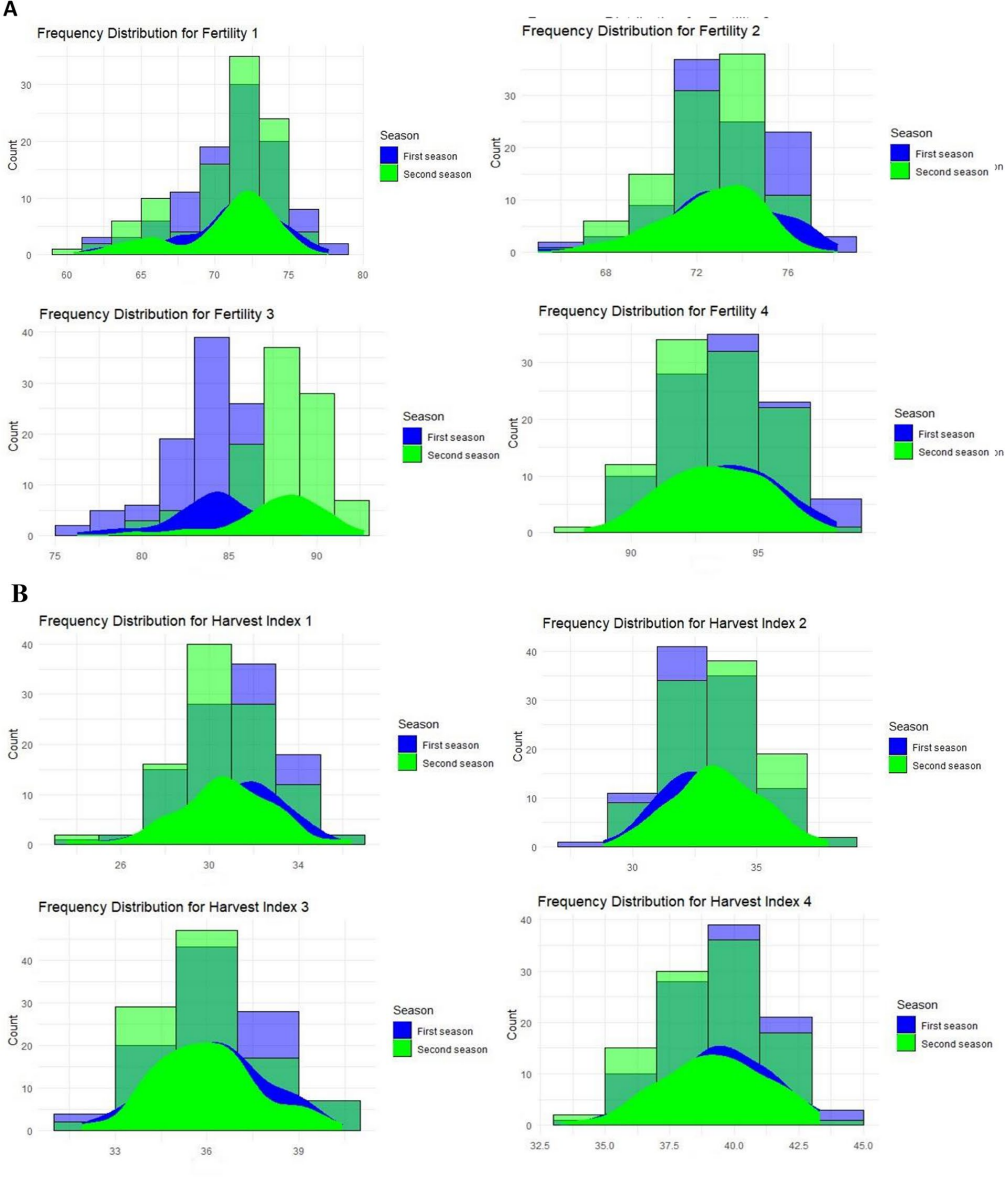
(**A**) Frequency distribution for a set of the studied genotypes for spikelet fertility (%), for the four different sowing dates (І, ІІ, ІІІ, and ІV) over the two growing seasons. (**B**) Frequency distribution for a set of the studied genotypes for harvest index (%), for the four different sowing dates (І, ІІ, ІІІ, and ІV) over the two growing seasons

Spikelet sterility was noticeable for SD1 and SD2, when low-temperature stress coincided with the reproductive period, with many of the investigated genotypes exhibiting 30% sterility (Fig. 4AI and II). The most tolerant genotype for SD2 had slightly more than 20% sterility. For the SD3, several genotypes were found to have low spikelet sterility, some with less than 20% sterility. However, most genotypes, comprising both cold-tolerant and -sensitive genotypes, exhibited less than 10% sterility for SD3 and SD4 (Fig. 4AIII and IV). Twelve genotypes planted on SD1 were tolerant to low temperatures, recording fertility percentages of more than 72%. The best genotypes were Sakha106, Reiho, and Giza 172 (Table S5 and Table S6). On SD2, fifteen genotypes showed more than 77% fertility percentages, including the tolerant check variety Giza 177 (Table S5 and Table S6). On the other hand, the most sensitive genotypes were IR 11 K 305 A and IR 83,106-B-B-2 (Table S5 and Table S6).

#### Harvest index (H1)

About 10% of the studied genotypes for the SD1 had harvest index percentage values ranging from 28.0 to 33.0% (Fig. 4BI). More than 10% of the genotypes had HI % ranging from 32.0 to 36.0% the reproductive stage (SD2), as opposed to SD3 and SD4, (Fig. 4BII). More than 15% of the genotypes had HI% of at least 35.0% and 38.0% for SD3 and SD4, respectively, which were not subjected to cold stress (Fig. 4BIII and IV). The GZ9626-2-1-3-2, GZ6903-1-2-2-1, and HR 20654-54-3-2 genotypes had HI% of 30% or more for all SDs (Table S6 and Table S8). For SD1, which coincided with cold stress, eleven genotypes showed HI% of 25% or less, including the sensitive variety IR 83,106-B-B-2 (Table S3 and Table S4). On the other hand, ten genotypes showed HI% values of more than 32%, including the tolerant variety Giza 177 on SD2 (Table S7 and Table S8).

#### Grain yield/plant

**Fig. 5:**
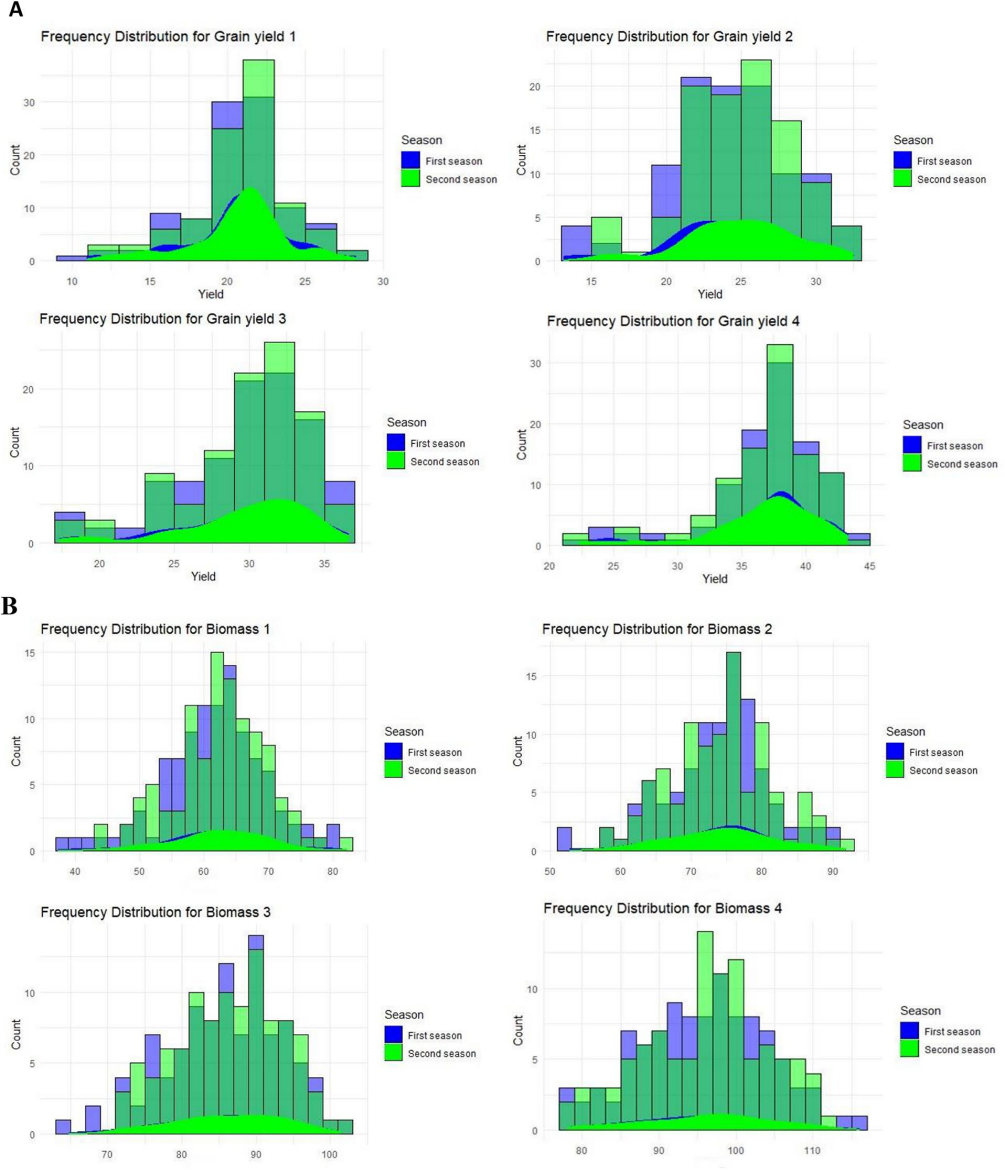
(**A**) Frequency distribution of a set of the studied genotypes for grain yield/plant (g), for the four different sowing dates (І, ІІ, ІІІ, and ІV) over the two growing seasons. (**B**) Frequency distribution of a set of the studied genotypes for biomass (g), for the four different sowing dates (І, ІІ, ІІІ, and ІV) over the two growing seasons

Grain yields were effectively decreased for plants sown on SD1 compared with the plants sown on SD2-SD4 because cold stress happened during the reproductive stage. More than 30% of the SD1 genotype produced only 20 g/plant (Fig. 5AI). Yields generally increased for SD2, with about 20% of genotypes yielding over 25 g/plant (Fig. 5AII) and many surpassing 30 g/plant for SD3 (Fig. 5AIII). On SD1, the coldest sowing date, only Sakha104, Giza 176, and Sakha107 yielded more than 25 g per plant (Table S6 and Table S8). For SD1, which coincided with cold stress, ten genotypes showed less than 20 g of grain yield per individual plant, including the sensitive genotype IR 83,106-B-B-2 (Table S6 and Table S8). On the other hand, six genotypes showed more than 28 g of grain yield per plant for SD2 when cold stress occurred, including the tolerant check genotype Giza 177 (Table S7 and Table S8).

#### Biomass

The biomass production was lower for SD1 than for SD2-SD4 (Table S4). About 20% of the genotypes produced biomass of 70 g/plant on SD1, while more than 30% produced biomass of 80 g/plant on SD2 (Fig. 5BII). For SD3, about 20% of the genotypes had a biomass of 90 g/plant (Fig. 5BII). For SD1, the coldest sowing date, Sakha104, Sakha107, and Sakha102 were the only genotypes that produced more than 70 g of biomass per plant (Table S4). During SD1, which experienced cold stress, 14 genotypes yielded 60 g or less per plant, including IR 83,106-B-B-2 (Table S4). In contrast, during SD2, also under cold stress conditions, ten genotypes yielded over 77 g of biomass per plant, including the tolerant check genotype Giza 177 (Table S4).

### Interrelationships among evaluated genotypes and measured traits at different dowing dates (SD)

**Fig. 6:**
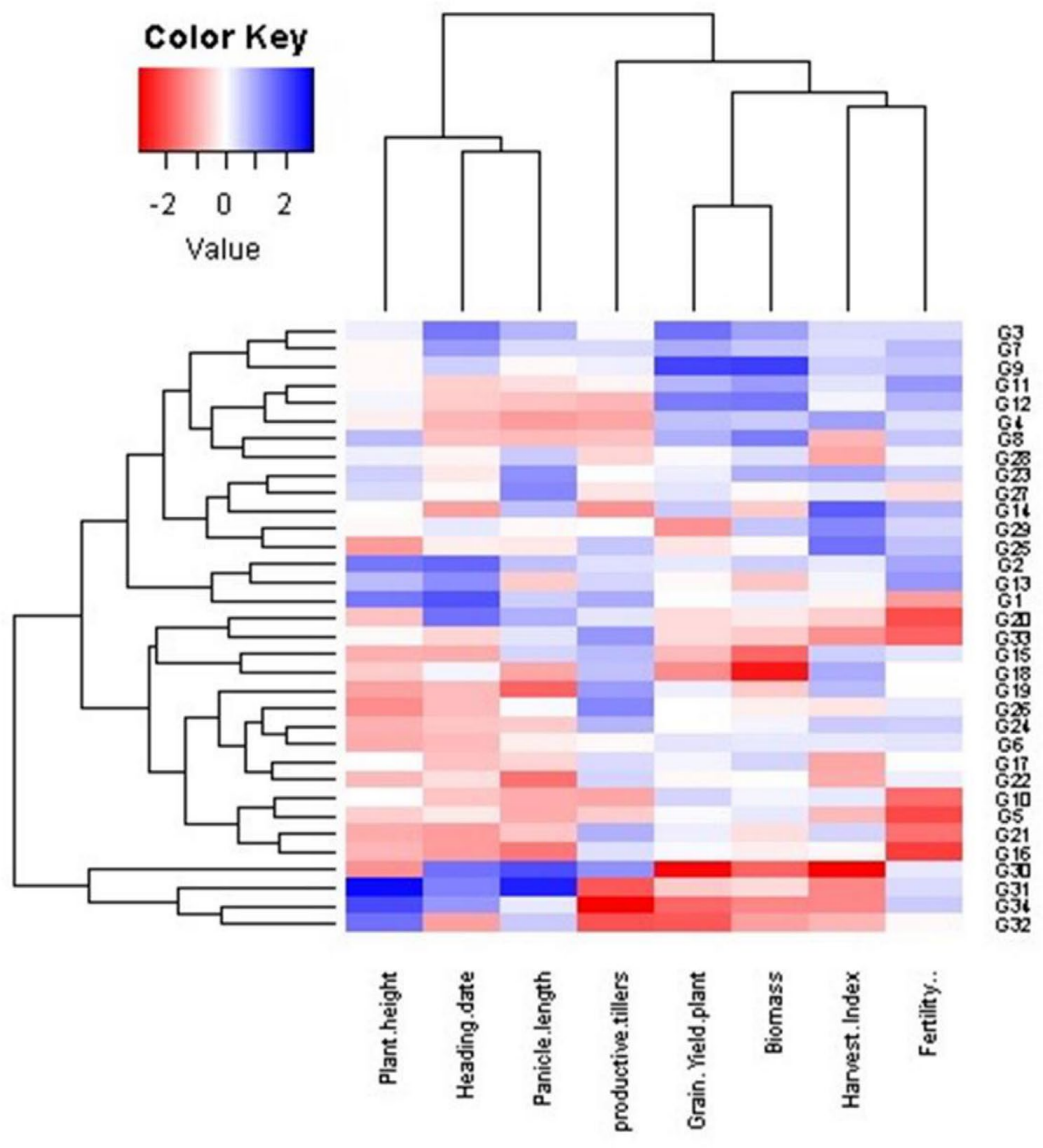
Heatmap and hierarchical clustering divide the evaluated 34 rice genotypes into different clusters based on the studied traits. Red and blue colors imply low and high values for the corresponding studied parameters at the first sowing date

**Fig. 7:**
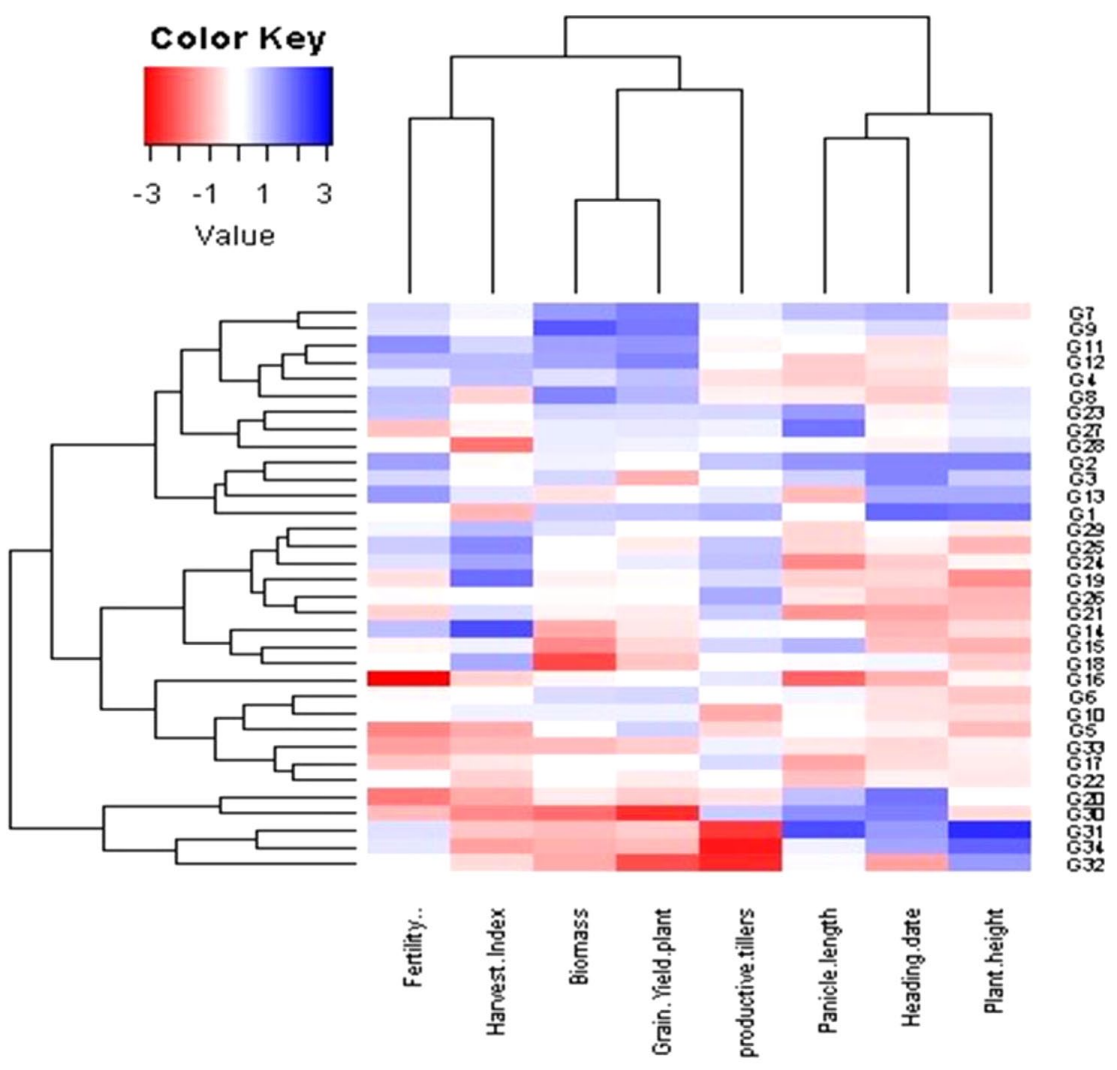
Heatmap and hierarchical clustering divided the evaluated 34 rice genotypes into different clusters based on the studied traits. Red and blue colors imply low and high values for the corresponding studied parameters at the second sowing date

**Fig. 8:**
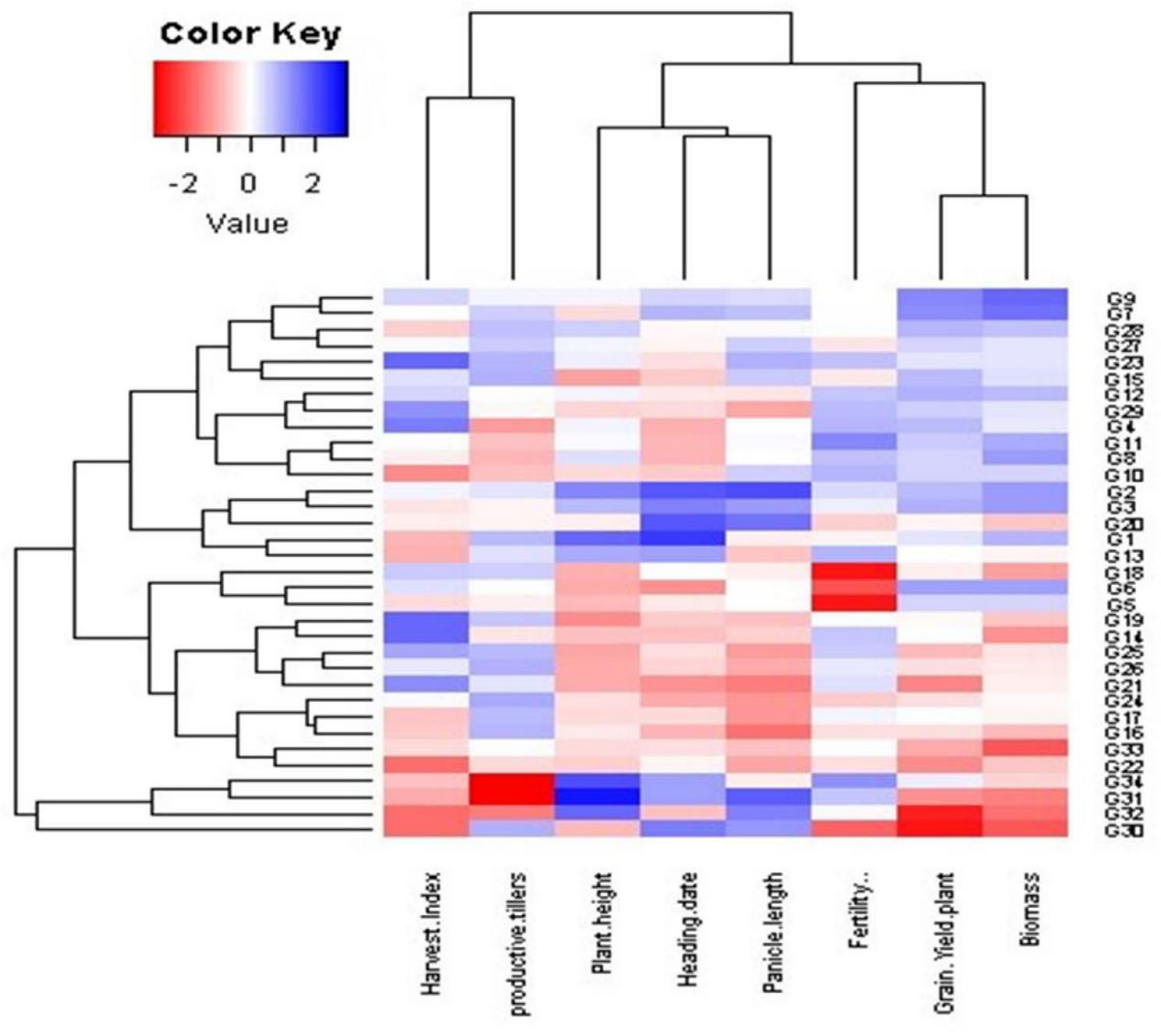
Heatmap and hierarchical clustering divide the evaluated 34 rice genotypes into different clusters based on the studied traits. Red and blue colors imply low and high values for the corresponding studied parameters at the third sowing date

**Fig. 9:**
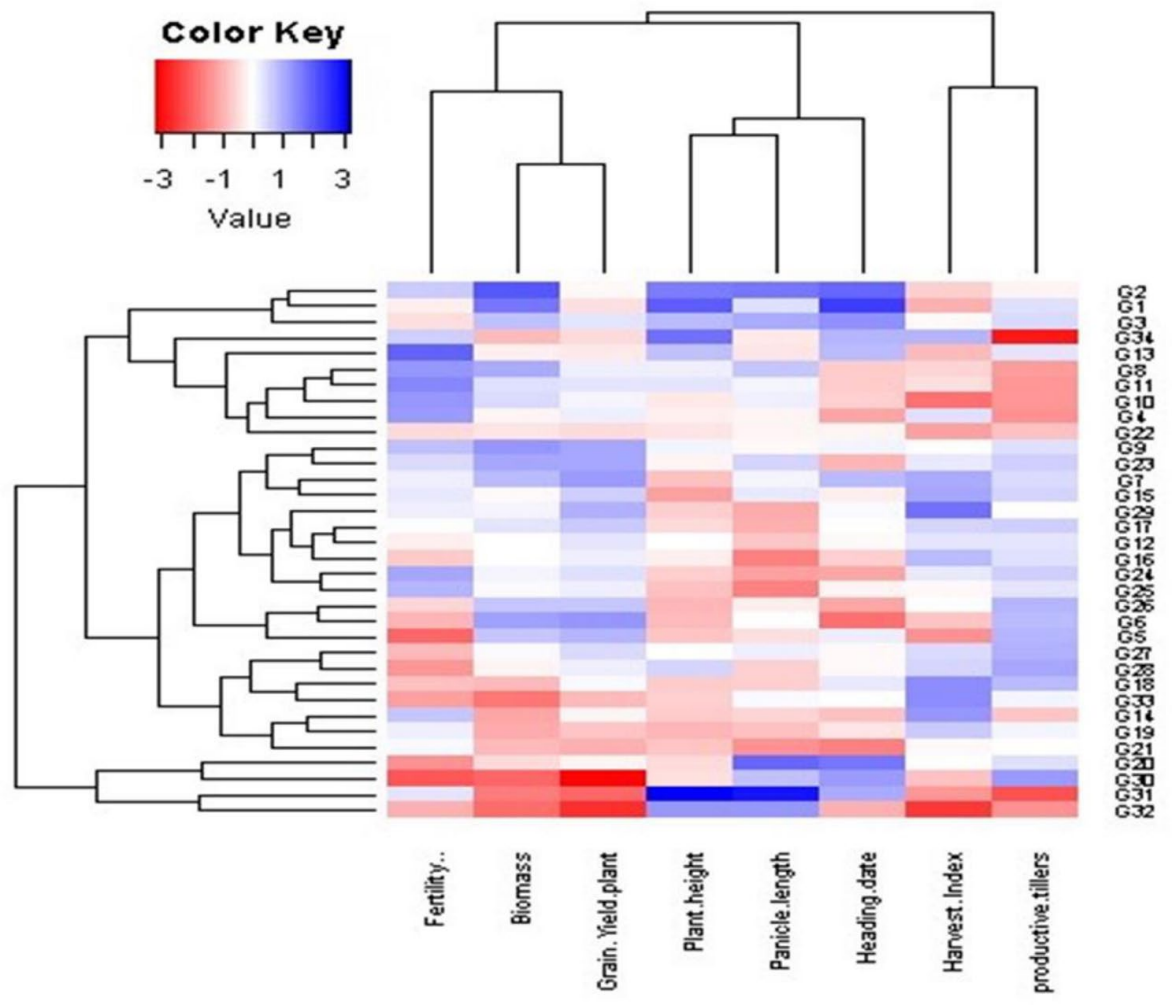
Heat map and hierarchical clustering divide the evaluated 34 rice genotypes into different clusters based on the studied traits. Red and blue colors imply low and high values for the corresponding studied parameters at the fourth sowing date

**Fig. 10:**
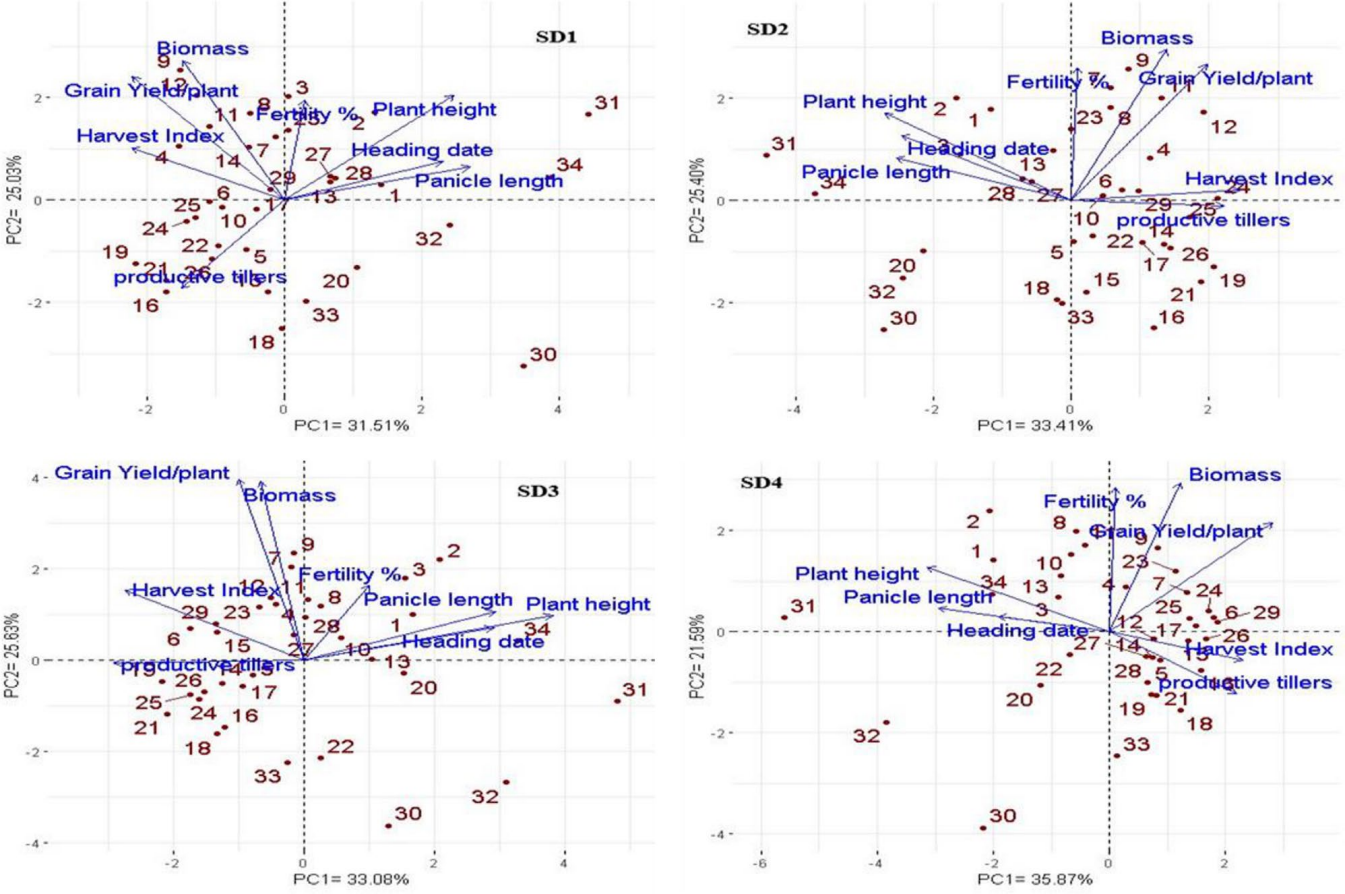
Biplot of PCA for the estimated 34 rice genotypes to explore the association among the studied traits at four different sowing dates; the first sowing date (**SD1**), the second sowing date (**SD2**), the third sowing date (**SD3**) and the fourth sowing date (**SD4**)

Principal component analysis (PCA) was employed to evaluate the relationship between the examined traits across various sowing dates and genotypes (Fig. 10). These findings align with the outcomes observed in the heatmap and hierarchical clustering analyses (Figs. 6, 7 and 8, and 9). The PC-biplot displays characters that exhibit strong positive associations as vectors closely positioned to one other. In contrast, characters that are almost opposite (at 180°) indicate a very negative relationship. As a result, a robust positive correlation has been shown between them.

#### First sowing date (SD1)

The multivariate analysis implied that the first two PCAs explained 56.54% of the total variation (Fig. 10). PC 1 comprises plant height, heading date, and panicle length. Genotypes 31 and 34 were positively associated with the previous traits and were situated oppositely to productive tillers. Otherwise, genotypes 16, 18, 19, 21, 22, 24, and 26 were positively associated with productive tillers and negatively correlated with plant height, heading date, and panicle length. In PC2, genotypes 4, 7, 8, 9, 11, and 12 positively correlated with grain yield, harvest index, and biomass, while genotypes 30 and 32 showed negative correlations. Hierarchical clustering based on morphological traits grouped genotypes into three clusters (Fig. 6). Genotypes 31, 32, and 34 had higher plant height, heading date, and panicle length but lower values of productive tillers, grain yield, harvest index, and biomass. The second cluster generally exhibited lower plant height, heading date, and panicle length. Meanwhile, genotypes 3, 7, and 9 consistently excelled across all traits, especially in grain yield and biomass.

#### Second sowing date (SD2)

The first two PCAs accounted for 58.81% of total variability, with PCA1 explaining 33.41% and PCA2 25.40%. PCA1 included grain yield per plant and biomass, with genotypes 4, 7, 8, 9, 11, and 12 positively associated, and genotypes 20, 30, and 32 negatively associated. PCA2 comprised plant height, heading date, and panicle length, with genotypes 31 and 34 linked to these traits and opposite productive tillers. Genotypes 16, 17, 19, 21, and 26 were positively associated with productive tillers and negatively with plant height, heading date, and panicle length. Heatmap and hierarchical clustering divided genotypes into three clusters (Fig. 7). Genotypes 20, 30, 31, 32, and 34 had high plant height, heading date, and panicle length but low productive tillers, grain yield, harvest index, and biomass. The second cluster showed mixed values, while the third cluster, especially genotypes 2, 7, and 9, excelled in all parameters, particularly grain yield, harvest index, fertility percentage, and biomass.

#### Third sowing date (SD3)

The third sowing date, close to the expected date in April, is depicted in the PCA plot of morphological parameters (Fig. 10), with PC1 and PC2 accounting for 58.71% of total variance. PC1 includes plant height, heading date, panicle length, and fertility percentage. Genotypes 2, 3, and 34 were positively associated with these traits but opposed to productive tillers, while genotypes 16, 18, 21, 24, and 33 showed negative correlations. PC2 positively associated grain yield per plant, harvest index, and biomass with genotypes 6, 7, 9, 11, 12, and 29, and negatively with genotypes 30 and 32. Hierarchical clustering divided genotypes into three clusters (Fig. 8). Genotypes 30 and 31 had high plant height, heading date, and panicle length but low fertility percentage, grain yield, harvest index, and biomass. The second cluster showed slightly lower plant height, heading date, and panicle length values. Genotypes 2, 9, and 23 performed best across all traits, with notably high grain yield and biomass.

#### Fourth sowing date (SD4)

For the expected sowing date (May), the first two PCAs accounted for 58.81% of the total variability, with PCA1 explaining 33.41% and PCA2 25.40% (Fig. 10). PC1 included grain yield per plant, fertility percentage, and biomass. Genotypes 4, 6, 7, 8, 9, 23, 24, and 29 were positively associated with these traits, while genotypes 20, 30, and 32 were negatively associated. PC2 comprised plant height, heading date, and panicle length, with genotypes 1, 2, 31, and 34 positively associated. Conversely, genotypes 5, 16, 18, 19, 21, 27, 28, and 33 were positively associated with productive tillers and harvest index but negatively with plant height, heading date, and panicle length. Hierarchical clustering divided the genotypes into three clusters (Fig. 9). Genotypes 30, 31, and 32 had high plant height, heading date, and panicle length but low productive tillers, grain yield, harvest index, fertility percentage, and biomass. The second cluster showed high grain yield, harvest index, fertility percentage, productive tillers, and biomass, with low plant height and heading date. The third cluster had high plant height and heading date but low harvest index and productive tillers, especially genotypes 1, 2, 3, 34, 11, and 13.

## Discussion

Rice, which originates from tropical and subtropical climates, is sensitive to low temperature. Cold stress significantly affects the growth and development of rice plants and limiting production in many countries worldwide [7]. Thus, the world requires continuous efforts to understand and adapt to environmental challenges and to develop new varieties for sustainable crop production. Intraspecific genetic variability for cold tolerance has been reported in rice, where *Japonica* cultivars have higher cold tolerance levels than *Indica* cultivars [22].

The identification of novel genetic resources for cold tolerance is of utmost importance in facilitating breeders’ development of cold-tolerant rice cultivars, specifically targeting temperate areas. To develop the resilient rice varieties capable of maintaining productivity in regions prone to low-temperature stress, it is crucial to carefully choose and assess diverse rice germplasms from tolerant rice line. These germplasms should be subjected to cold stress at various developmental stages such as during booting, and flowering stages at *Japonica* rice [22]. It is also essential to conduct crossbreeding with a wide range of elite cultivars from various nations [23]. Nevertheless, there is a scarcity of genetic resources that may be used to enhance the cold tolerance of temperate Japonica rice varieties. In this context, several mild Japonica breeding lines have included cold tolerance genes from the Silewah, Lambayque 1, and Padi Labou Alumbis cultivars [24].

Evaluating materials for freezing tolerance is challenging, particularly in ensuring accuracy and reliability. Therefore, using replicable phenotyping techniques and appropriate statistical analyses is essential for assessing cold tolerance during the reproductive phase.

### Analysis of variance (ANOVA)

The analysis of variance (ANOVA) findings demonstrated statistically significant variations among the 34 genotypes examined for all the evaluated attributes in the current research. Significant mean squares for genotypes were observed across all parameters and sowing dates, with interactions among genotypes showing varied responses to different SDs. Similar to our study, Shu al. [22] found that 23 elite rice cultivars sourced from 8 temperate countries exhibited differences in cold tolerance between indica and japonica rice. This study also revealed significant variations in attributes attributed to genotypes and SDs. These results indicating for high variances between genotypes in addition to effective treatments by SDs as a different temperature under Egyptian conditions. However, the effect of growing season were little. In line of with our results, growing seasons impact on rice was not highly significant under Egypt conditions [24]. The genotypes showed diverse genetic compositions, with SD significantly impacting their performance. The existence of such ample genetic diversity may be attributed to the different origins of the present population under investigation and environmental variables that impact phenotypic performance [25]. Thus, our research highlighted ample opportunity to identify genotypes with favorable agro-physiological traits.

### Interaction between genotype and environment on morphological traits and grain yield

Seasonal temperature fluctuations in the north Delta from February to May indicating the requirement of rice varieties resilient to low-temperature stress. This study found significant differences in genotype performance across SDs, aligning with past research distinguishing cold-tolerant and -sensitive genotypes [24, 26, 27]. It highlights the importance of genetic diversity in developing new genotypes [28] that enhance productivity and adaptability to low-temperature stress. Typically, the yield will exhibit greater magnitude when the conventional sowing date aligns with or experiences little cold stress throughout the vegetative phase, followed by a sowing date in April. As a consequence of the decrease in temperature, grain yields reached their minimum point in the month of March.

### Effect of cold stress on morphological traits and grain yield

Understanding these morphological responses to cold stress is crucial for developing strategies to enhance rice tolerance to low temperatures. According to our results, the occurrence of cold stress during the reproductive phase resulted in a reduction in panicle length, grain HI and grain production. Additionally, cold stress increased the duration of the heading date and spikelet sterility. The influence of cold stress on spikelet fertility is more pronounced because of its induction of microspore degeneration and sterility during micro-sporogenesis [29]. Furthermore, the extensive range of genotypes exhibiting adverse panicle exertions indicates that cold stress hampers the emergence of panicles from the protective sheath.

Based on these traits we, first, selected six rice genotypes Sakha104, Giza176, Sakha107, Sakha102, Sakha106, Giza 177 (genotypes 9, 3, 12, 8, 11 and 4 and tolerant check, repectivly). It is worth noting that all of these genotypes belonged to the Japonica group. The findings align with prior research [22], indicating spikelet reproduction rates exceeding 71% under controlled greenhouse conditions for Giza 177 (Egypt), Avangard and Mustaqillik (Uzbekistan), and Jinbu and Jungan (Korea), highlighting their potential as genetic resources to enhance cold resistance and spikelet fertility in japonica rice.

Following exposure to cold stress during the reproductive stage, the sex genotypes exhibited satisfactory yields and favorable cold resistance features. PCA analysis indicated that the genotypes CIASEM (30), Carola (32), I Geo Tze (33), WOMBAT (34), IR 83,106-B-B-2 (the sensitivity check) (20), and MILYANG 240 (18) shown favorable performance as sensitive genotypes under low-temperature stress conditions. These genotypes above were classified as members of the indicia group. They exhibited diminished grain production per plant, reduced fertility percentages, suboptimal biomass, and decreased HI for SD1-SD4. In line with our study, indica rice exhibits comparatively lower cold tolerance at [22]. Consistently, planting rice varieties vulnerable to cold stress at an early stage reduced yield, potentially due to the adverse effects of cold stress during the reproductive phase (13, 14). Nonetheless, it is essential to choose genotypes that exhibit high yields and minimum delay in maturity. The findings indicate that cold stress leads to a drop-in grain yield and an increase in the percentage of partly-filled grains. It is worth noting that partially filled grains are more likely to break during milling. Consequently,

Cold stress during the vegetative phase could be employed as a means to enhance rice productivity. Cold stress during the vegetative stage on SD3 produced a set of genotypes that exhibited favorable yields, moderate to short growth durations, and significant biomass accumulation. On the other hand, cold stress lead to a drop-in grain yield and an increase in partly-filled grains%. It is worth noting that partially filled grains are more likely to break during milling. The genotypes (Giza 179 (indica/japonica type) (6), Sakha 102 (8), Sakha 104 (9), Sakha 106 (11), Sakha 107 (12), GZ 9730-1-1-1-1 (23), and GZ 1368-S-5-4 (28) (Indica type)) showed considerable potential for double cropping within the desired northern Delta region. The parental genotypes, Giza178 and Giza179, included in this research, are classified as indica rice cultivars and exhibit significant genetic divergence from other members of the indicia group. These two cultivars demonstrated excellent productivity and short growth cycles, along with commendable morphological traits during the vegetative phase. Hence, selecting rice genotypes tolerant to cold stress is vital for enhancing yields in early-sown indica genotypes [15, 22, 23].

The donor parent of cold tolerance is crucial, exemplified by genotypes 16 (IR 11 K 305 A) and 27 (IET 1444) in the indica type. These genotypes exhibit favorable traits like high yields, early to medium durations, and strong spikelet fertility, maintaining cold tolerance through the vegetative stage. However, they show reduced fertility rates, average yields, and fewer productive tillers due to cold stress during reproduction, suitable mainly for planting during the second sowing date. This observation deepens understanding of abiotic stress effects on rice growth, prompting new breeding strategies. Identifying rice cultivars resilient to cold stress is essential to enhance yields for early-sown indica genotypes [14, 17, 30]. In addition, prior research shown that decreases in yield traits might lead to the development of vegetative stages more resistant to low temperatures [31]. However, the reduction in growth during the later stages may be comparable to or less than that in the terminal growth stage if cold conditions occur, as there is a higher chance of recovery growth towards the end of the reproductive period after blooming [32].

### The relationship between morpho-physiological characteristics and grain yield under cold-tolerance stress conditions

Studying these relationships can provide insights into how different varieties respond to cold stress, potentially identifying genetic traits that enhance yield stability in challenging environmental. The reproductive stage is susceptible to low-temperature stress, making it one of the most vulnerable development phases. In this regard the detrimental effects of cold stress on rice output are mostly seen during the blooming and grain-filling phases including floral fertility [16]. This is attributed to the adverse impact of cold stress on many physiological systems in rice, including floral fertility. Most genotypes examined in this research exhibited a drop in spikelet fertility under stressful conditions. There was a strong correlation between environmental pressures experienced throughout the reproductive period and a significant decrease in fertility % [7, 33, 34]. Previous studies have shown that low temperatures during the vegetative stage of rice cultivation have been found to extend the growth period [7, 35]. In contrast, cold stress experienced during the reproductive phase leads to panicle sterility and a decrease in grain production and yield [7, 10]. Cold stress also impacted the extension of panicles, leading to further loss in yield due to the increased sterility of incorrectly expanded panicles. Rice grains covered by flag leaf sheaths at maturity often exhibit sterility [42]. The extent of harm related to cold stress is contingent upon the developmental stage and the severity of the cold experience [12].

Subjecting rice plants to cold stress inhibited their development and reduced biomass due to restricted water absorption, limited nutrient availability, and disrupted biochemical processes. This led to decreased net photosynthesis rates and slowed cell division and elongation, ultimately impairing overall plant growth [36].

The extent of correlation between characteristics is also essential, particularly in complicated qualities like yield [37, 38]. We demonstrated a correlation between early development and increased productivity in the face of cold stress during the reproductive stage. A notable association was seen between grain production and biomass, tillering ability, and spikelet fertility in instances, when cold stress only transpired during the vegetative stage, as opposed to the mature stage. Farmers can benefit from selecting genotypes showing cold tolerance, especially for early planting, based on associations between genotypes and SD. Previous studies indicate that rice genotypes with more effective tillers per plant correlate with higher grain yields [39]. The selection criteria for phenotyping cereal crops for abiotic stress tolerance, such as cold tolerance [40] and low- or high-temperature tolerance [41, 42], has included using a comparable response indicator, such as the harvest index. Farmers may effectively prepare their fields and complete post-harvest tasks before the primary growing season in July by engaging in planting efforts during the cold period in late February.

Overall, applying cold stress in the vegetative phase may serve as a means to enhance rice productivity. However, it is crucial to carefully choose rice types that exhibit both high yields and little delay in maturity.

## Conclusions

This study provides essential data for enhancing the cold tolerance of rice through breeding programs. It demonstrates that exposure to low temperatures during the reproductive phase prolongs crop maturation, reduces panicle length, spikelet fertility, and grain yield, significantly impacting production, especially in sensitive varieties. In Egypt, genotypes like Giza176, Sakha104, and Sakha107 are recommended for early cultivation (March 1st), while Giza 179, Sakha101, Sakha104, and GZ 9730-1-1-1-1 are suitable for average cultivation (May 1st), with Sakha104 being particularly versatile for both. Future perspectives include selecting rice varieties for double cropping in Egypt’s Northern Delta. Incorporating cold-resistant varieties from colder regions like Hungary and Russia, though not covered in this study, would enrich future research. Comparing their resilience and adaptability across different environmental conditions could further enhance our understanding and selection of optimal rice cultivars.

### Electronic supplementary material

Below is the link to the electronic supplementary material.

Supplementary Material 1

## Data Availability

The authors confirm that the data supporting the findings of this study are available within the supplementary materials.
