# Supplementary material for: Mitigating cold stress in rice: a study of genotype performance and sowing time
Source: BMC Plant Biol. 2024 Jul 26;24:713. doi: 10.1186/s12870-024-05423-8 (PMC11282823; doi:10.1186/s12870-024-05423-8)
Supplement: Supplementary file 1 — Supplementary Material 1 [file 12870_2024_5423_MOESM1_ESM.docx]

Table S1. Mean performance of heading date and plant height traits over two growing seasons

| No. | Genotypes | Heading date (days) | | | | | Plant height (cm) | | | | |
| --- | --- | --- | --- | --- | --- | --- | --- | --- | --- | --- | --- |
|  |  | SD1 | SD2 | SD3 | SD4 | Mean | SD1 | SD2 | SD3 | SD4 | Mean |
| 1 | Giza 171 | 144.12 | 135.27 | 129.7 | 119.49 | 132.145 | 109.25 | 115.98 | 120.83 | 132.22 | 119.57 |
| 2 | Giza 172 | 141.52 | 131.87 | 126.6 | 115.88 | 128.9675 | 109.27 | 114.12 | 117.25 | 128.17 | 117.2025 |
| 3 | Giza 176 | 139.67 | 131.96 | 122.56 | 112.38 | 126.6425 | 95.03 | 105.67 | 111.55 | 119.97 | 108.055 |
| 4 | Giza 177 | 111.73 | 110.38 | 101.46 | 95.17 | 104.685 | 90.25 | 98.67 | 105.03 | 108.56 | 100.6275 |
| 5 | Giza 178 | 118.17 | 112.71 | 107.42 | 104.22 | 110.63 | 86.88 | 87.75 | 95.63 | 102.95 | 93.3025 |
| 6 | Giza 179 | 112.06 | 110.96 | 98.71 | 90.83 | 103.14 | 82.2 | 89.52 | 93.88 | 101.73 | 91.8325 |
| 7 | Sakha 101 | 134.47 | 126.66 | 117.4 | 108.96 | 121.8725 | 91.73 | 92.28 | 98.83 | 102.2 | 96.26 |
| 8 | Sakha 102 | 112.63 | 108.65 | 102 | 98.42 | 105.425 | 101.4 | 103.25 | 107.55 | 113.4 | 106.4 |
| 9 | Sakha 104 | 127.88 | 121.96 | 113.95 | 103.79 | 116.895 | 91.47 | 97.62 | 104.85 | 112.43 | 101.5925 |
| 10 | Sakha 105 | 113.6 | 111.04 | 104.35 | 98.5 | 106.8725 | 92.35 | 92.05 | 99.25 | 107.8 | 97.8625 |
| 11 | Sakha 106 | 114.72 | 110.63 | 102.16 | 98.2 | 106.4275 | 91.7 | 95.83 | 104.13 | 114.97 | 101.6575 |
| 12 | Sakha 107 | 114.3 | 111.58 | 107.15 | 102.04 | 108.7675 | 94.28 | 95.05 | 105.05 | 111.63 | 101.5025 |
| 13 | Reiho | 136.64 | 127.54 | 119.33 | 108.99 | 123.125 | 101.05 | 109.3 | 113.02 | 119.42 | 110.6975 |
| 14 | HR 20654-54-3-5 | 109 | 105.79 | 103.2 | 97.45 | 103.86 | 92.88 | 91.92 | 96.28 | 104.18 | 96.315 |
| 15 | IR 68333-R-R-B-19 | 109.74 | 107.46 | 104.23 | 101.4 | 105.7075 | 82.75 | 86.67 | 92.4 | 98.15 | 89.9925 |
| 16 | IR 11K 305A | 107.84 | 105.46 | 102.25 | 98.46 | 103.5025 | 83.67 | 94.82 | 100.94 | 108.77 | 97.05 |
| 17 | IR 12K 269 | 113.06 | 110.06 | 106.02 | 102.7 | 107.96 | 93.23 | 93.92 | 99.23 | 105.95 | 98.0825 |
| 18 | MILYANG 240 | 122.97 | 118.93 | 109.62 | 104.85 | 114.0925 | 85.98 | 89.63 | 94.48 | 103.97 | 93.515 |
| 19 | Korea 14 | 112.04 | 109.92 | 104.48 | 100.63 | 106.7675 | 80.77 | 82.83 | 90.15 | 100.92 | 88.6675 |
| 20 | IR 83106-B-B-2 | 140.13 | 133.67 | 126.6 | 114.81 | 128.8025 | 85.08 | 96.58 | 101.27 | 105.65 | 97.145 |
| 21 | SKC 2015-1 | 108.28 | 103.79 | 98.67 | 92.18 | 100.73 | 82.27 | 87.8 | 93.78 | 103.4 | 91.8125 |
| 22 | SKC 2015-2 | 116.76 | 112.49 | 108.48 | 101.88 | 109.9025 | 84.17 | 92.85 | 97.58 | 107.12 | 95.43 |
| 23 | GZ 9730-1-1-1-1 | 118.23 | 112.71 | 106.1 | 96.38 | 108.355 | 98.58 | 102.62 | 105.33 | 109.07 | 103.9 |
| 24 | GZ 9730-1-1-3-2 | 113.2 | 108.16 | 101.1 | 95.21 | 104.4175 | 82.67 | 95.17 | 99.57 | 104.78 | 95.5475 |
| 25 | GZ 9626-2-1-3-2 | 119.27 | 112.5 | 106.06 | 101.68 | 109.8775 | 80.48 | 87.38 | 93.03 | 102.62 | 90.8775 |
| 26 | GZ 6296-12-1-2-1 | 111.93 | 107.71 | 105.06 | 94.97 | 104.9175 | 78.48 | 87.4 | 93.68 | 100.9 | 90.115 |
| 27 | IET 1444 | 120.2 | 114.21 | 108.51 | 102.05 | 111.2425 | 97.48 | 101.23 | 105.08 | 111.5 | 103.8225 |
| 28 | GZ 1368-S-5-4 | 119.93 | 112.96 | 109.02 | 102.22 | 111.0325 | 94.97 | 104.05 | 109 | 117.12 | 106.285 |
| 29 | GZ 6903-1-2-2-1 | 124.44 | 117.57 | 105.89 | 103.51 | 112.8525 | 91.65 | 93.37 | 98.4 | 104.67 | 97.0225 |
| 30 | CIASEM | 140.13 | 132.79 | 122.67 | 111.59 | 126.795 | 79.4 | 91.53 | 95.6 | 106.53 | 93.265 |
| 31 | Sanakevelle | 137.87 | 129.38 | 119.5 | 110.17 | 124.23 | 122.32 | 123.83 | 130.83 | 145.02 | 130.5 |
| 32 | Carola | 108.37 | 103.48 | 103.33 | 96.08 | 102.815 | 109.8 | 111.6 | 120.78 | 124.63 | 116.7025 |
| 33 | I Geo Tze | 115.19 | 109.83 | 106.13 | 102.71 | 108.465 | 91.77 | 94.17 | 98.82 | 104.38 | 97.285 |
| 34 | WOMBAT | 135.17 | 128.17 | 119.51 | 109.19 | 123.01 | 115.12 | 117.67 | 124.28 | 129.98 | 121.7625 |
| LSD at 5% | | 0.66 | | | | | 0.57 | | | | |
| LSD at 1% | | 0.87 | | | | | 0.75 | | | | |

Table S2. Mean performance of heading date and plant height traits of each growing season and over two growing seasons (mean±SD).

|  |  | **Heading date (days)** | | | **Plant height (cm)** | | |
| --- | --- | --- | --- | --- | --- | --- | --- |
| No. | **Genotype Name** | **Y1** | **Y2** | **Y1+Y2** | **Y1** | **Y2** | **Y1+Y2** |
| 1 | Giza 171 | 131.1±9.5 | 133.1±9.2 | 132.1±9.4 | 66.5±62.8 | 83.3±61 | 74.9±61.9 |
| 2 | Giza 172 | 128.7±9.4 | 129.3±10 | 129±9.7 | 65±61.7 | 80.7±59.3 | 72.9±60.5 |
| 3 | Giza 176 | 126.6±11 | 126.6±10.4 | 126.6±10.7 | 60.5±57.3 | 76.1±55.8 | 68.3±56.6 |
| 4 | Giza 177 | 104.9±7.5 | 104.4±6.9 | 104.7±7.2 | 56.1±53.1 | 70.5±51.6 | 63.3±52.4 |
| 5 | Giza 178 | 110.6±5.9 | 110.7±5.5 | 110.7±5.7 | 51.9±48.9 | 65.1±47.4 | 58.5±48.2 |
| 6 | Giza 179 | 103.4±9.7 | 102.9±8.9 | 103.2±9.3 | 51.3±48.6 | 64.3±46.7 | 57.8±47.7 |
| 7 | Sakha 101 | 121.9±10.2 | 121.8±10 | 121.9±10.1 | 53.2±50.3 | 66.4±48.5 | 59.8±49.4 |
| 8 | Sakha 102 | 105.5±5.9 | 105.3±5.8 | 105.4±5.9 | 58.9±55.3 | 73.3±53.4 | 66.1±54.4 |
| 9 | Sakha 104 | 117.2±9.7 | 116.6±9.3 | 116.9±9.5 | 56.9±53.8 | 71.3±52.1 | 64.1±53 |
| 10 | Sakha 105 | 107.4±6.2 | 106.4±6.3 | 106.9±6.3 | 54.4±51.4 | 67.9±49.5 | 61.2±50.5 |
| 11 | Sakha 106 | 107.1±7 | 105.8±6.8 | 106.5±6.9 | 56.9±54 | 71.7±52.5 | 64.3±53.3 |
| 12 | Sakha 107 | 108.4±4.6 | 109.1±5.2 | 108.8±4.9 | 55.9±53 | 71.2±52.3 | 63.6±52.7 |
| 13 | Reiho | 123.5±10.7 | 122.8±10.7 | 123.2±10.7 | 61.3±57.9 | 77±56.2 | 69.2±57.1 |
| 14 | HR 20654-54-3-5 | 104±4.3 | 103.7±4.7 | 103.9±4.5 | 53.3±50.2 | 66.4±48.4 | 59.9±49.3 |
| 15 | IR 68333-R-R-B-19 | 105.8±3.2 | 105.7±3.7 | 105.8±3.5 | 50±47.2 | 62.9±45.7 | 56.5±46.5 |
| 16 | IR 11K 305A | 103.5±3.8 | 103.5±3.9 | 103.5±3.9 | 54.6±51.7 | 69.1±50.2 | 61.9±51 |
| 17 | IR 12K 269 | 108±4.1 | 107.9±4.4 | 108±4.3 | 54.5±51.4 | 67.7±49.5 | 61.1±50.5 |
| 18 | MILYANG 240 | 114.2±8.1 | 114±7.2 | 114.1±7.7 | 52.6±49.7 | 65.2±47.6 | 58.9±48.7 |
| 19 | Korea 14 | 106.7±5.2 | 106.8±4.3 | 106.8±4.8 | 49.4±46.9 | 62.4±45.6 | 55.9±46.3 |
| 20 | IR 83106-B-B-2 | 129±9.7 | 128.6±10 | 128.8±9.9 | 54±51.2 | 68.6±49.8 | 61.3±50.5 |
| 21 | SKC 2015-1 | 100.3±6 | 101.1±6.7 | 100.7±6.4 | 51.6±48.6 | 64.6±46.9 | 58.1±47.8 |
| 22 | SKC 2015-2 | 110.1±6.2 | 109.7±5.6 | 109.9±5.9 | 53.2±50.4 | 67.5±49.2 | 60.4±49.8 |
| 23 | GZ 9730-1-1-1-1 | 108.3±9.2 | 108.4±8 | 108.4±8.6 | 57.6±54.4 | 71.3±51.8 | 64.5±53.1 |
| 24 | GZ 9730-1-1-3-2 | 104.1±7.2 | 104.7±7.2 | 104.4±7.2 | 53.6±50.6 | 67.8±49 | 60.7±49.8 |
| 25 | GZ 9626-2-1-3-2 | 109.8±7.4 | 109.9±6.7 | 109.9±7.1 | 51.1±48.4 | 64.1±46.7 | 57.6±47.6 |
| 26 | GZ 6296-12-1-2-1 | 104.8±7 | 105.1±6.2 | 105±6.6 | 50.8±47.8 | 63.8±46.4 | 57.3±47.1 |
| 27 | IET 1444 | 111.2±7.1 | 111.3±7.2 | 111.3±7.2 | 57.8±54 | 71.4±52.1 | 64.6±53.1 |
| 28 | GZ 1368-S-5-4 | 111.1±7.6 | 111±6 | 111.1±6.8 | 59.5±56.2 | 74.5±54.2 | 67±55.2 |
| 29 | GZ 6903-1-2-2-1 | 113±10.3 | 112.7±7.7 | 112.9±9 | 54±50.7 | 66.9±48.5 | 60.5±49.6 |
| 30 | CIASEM | 126.7±11.9 | 126.9±10.7 | 126.8±11.3 | 52.8±50 | 66.4±48.6 | 59.6±49.3 |
| 31 | Sanakevelle | 124.6±11.4 | 123.9±10.5 | 124.3±11 | 72.2±68.6 | 90.4±66.2 | 81.3±67.4 |
| 32 | Carola | 102.4±4.7 | 103.3±4.7 | 102.9±4.7 | 64.6±61.2 | 81±59 | 72.8±60.1 |
| 33 | I Geo Tze | 108.6±5.5 | 108.4±4.4 | 108.5±5 | 54.5±51.4 | 66.9±48.8 | 60.7±50.1 |
| 34 | WOMBAT | 123.1±11 | 123±9.4 | 123.1±10.2 | 67.2±63.4 | 84±61.3 | 75.6±62.4 |

Table S3. Mean performance of panicle length and productive tillers traits over two growing seasons

| No. | Genotypes | Panicle length (cm) | | | |  | No. of productive tillers/plant | | | | |
| --- | --- | --- | --- | --- | --- | --- | --- | --- | --- | --- | --- |
|  |  | SD1 | SD2 | SD3 | SD4 | Mean | SD1 | SD2 | SD3 | SD4 | Mean |
| 1 | Giza 171 | 19.63 | 20.12 | 21.07 | 23.11 | 20.98 | 15.18 | 16.85 | 20.05 | 21.4 | 18.37 |
| 2 | Giza 172 | 19.87 | 22.27 | 24.6 | 24.94 | 22.92 | 13.68 | 16.35 | 18.65 | 19.83 | 17.13 |
| 3 | Giza 176 | 20.05 | 21.3 | 23.27 | 24.01 | 22.16 | 12.88 | 14.28 | 17.28 | 21.63 | 16.52 |
| 4 | Giza 177 | 17.37 | 19.17 | 21.43 | 22.28 | 20.06 | 10.1 | 13.05 | 14.52 | 16.68 | 13.59 |
| 5 | Giza 178 | 17.55 | 20.48 | 21.47 | 21.88 | 20.35 | 11.17 | 12.71 | 17.22 | 22.82 | 15.98 |
| 6 | Giza 179 | 18.65 | 20.63 | 21.5 | 22.39 | 20.79 | 12.47 | 13.95 | 17.88 | 22.55 | 16.71 |
| 7 | Sakha 101 | 19.5 | 21.56 | 22.53 | 22.68 | 21.57 | 13.8 | 15.38 | 19.42 | 21.6 | 17.55 |
| 8 | Sakha 102 | 17.85 | 19.63 | 21.47 | 23.54 | 20.62 | 10.87 | 13.52 | 15.28 | 16.95 | 14.16 |
| 9 | Sakha 104 | 18.78 | 20.65 | 22.1 | 22.38 | 20.98 | 13.2 | 14.75 | 18.18 | 21.38 | 16.88 |
| 10 | Sakha 105 | 17.6 | 19.95 | 22.27 | 22.79 | 20.65 | 10.13 | 11.45 | 15.75 | 16.93 | 13.57 |
| 11 | Sakha 106 | 18.38 | 20.15 | 21.53 | 22.73 | 20.7 | 12.27 | 13.62 | 15.72 | 16.8 | 14.6 |
| 12 | Sakha 107 | 17.92 | 19.15 | 20.87 | 21.41 | 19.84 | 10.6 | 14.59 | 17.75 | 21.27 | 16.05 |
| 13 | Reiho | 18.08 | 18.83 | 20.37 | 21.96 | 19.81 | 13.97 | 15.4 | 18.88 | 21.17 | 17.36 |
| 14 | HR 20654-54-3-5 | 19.88 | 20.05 | 20.53 | 21.71 | 20.54 | 9.53 | 14.82 | 16.77 | 18.33 | 14.86 |
| 15 | IR 68333-R-R-B-19 | 19.55 | 21.78 | 22.4 | 22.91 | 21.66 | 14.73 | 15.89 | 20.42 | 21.67 | 18.18 |
| 16 | IR 11K 305A | 16.8 | 17.4 | 18.88 | 20.17 | 18.31 | 13.67 | 15.49 | 20.25 | 21.37 | 17.7 |
| 17 | IR 12K 269 | 18.22 | 18.48 | 19.5 | 21.08 | 19.32 | 13.8 | 15.82 | 20.07 | 21.97 | 17.92 |
| 18 | MILYANG 240 | 17.52 | 20.58 | 21.07 | 21.59 | 20.19 | 14.47 | 14.15 | 19.4 | 22.65 | 17.67 |
| 19 | Korea 14 | 16.47 | 19.25 | 20.27 | 21.31 | 19.33 | 15.53 | 15.72 | 19.63 | 20.7 | 17.9 |
| 20 | IR 83106-B-B-2 | 20.13 | 21.58 | 23.98 | 25.24 | 22.73 | 13.53 | 12.99 | 17.4 | 21.47 | 16.35 |
| 21 | SKC 2015-1 | 17.95 | 18.25 | 19.07 | 20.53 | 18.95 | 14.97 | 16.24 | 18.72 | 20.23 | 17.54 |
| 22 | SKC 2015-2 | 16.72 | 19 | 19.8 | 22.24 | 19.44 | 14 | 14.54 | 16.45 | 18.27 | 15.82 |
| 23 | GZ 9730-1-1-1-1 | 20.6 | 22.15 | 22.87 | 23.28 | 22.23 | 12.6 | 15.88 | 20.15 | 21.9 | 17.63 |
| 24 | GZ 9730-1-1-3-2 | 18.07 | 18.07 | 19.47 | 20.81 | 19.11 | 14.77 | 16.46 | 20.45 | 21.9 | 18.4 |
| 25 | GZ 9626-2-1-3-2 | 18.55 | 19.32 | 19.6 | 20.24 | 19.43 | 14.33 | 16.35 | 20.08 | 21.2 | 17.99 |
| 26 | GZ 6296-12-1-2-1 | 19.05 | 19.58 | 19.87 | 22.14 | 20.16 | 16.2 | 17.18 | 20.28 | 22.68 | 19.09 |
| 27 | IET 1444 | 20.8 | 22.85 | 22.33 | 22.84 | 22.21 | 11.8 | 15.16 | 19.45 | 22.88 | 17.32 |
| 28 | GZ 1368-S-5-4 | 19.72 | 20.55 | 21.53 | 21.62 | 20.86 | 11.4 | 14.72 | 19.83 | 23.23 | 17.3 |
| 29 | GZ 6903-1-2-2-1 | 18.75 | 19.32 | 19.77 | 20.98 | 19.71 | 12.8 | 14.73 | 17.45 | 20.37 | 16.34 |
| 30 | CIASEM | 21.62 | 22.32 | 23.33 | 23.64 | 22.73 | 15.8 | 16.16 | 20.35 | 23.57 | 18.97 |
| 31 | Sanakevelle | 22.4 | 23.42 | 24.37 | 26.71 | 24.23 | 7.87 | 8.31 | 9.45 | 14.63 | 10.07 |
| 32 | Carola | 19.72 | 20.6 | 23.67 | 24.41 | 22.1 | 7.77 | 7.81 | 13.5 | 16.73 | 11.45 |
| 33 | I Geo Tze | 19.31 | 19.58 | 20.22 | 22.61 | 20.43 | 15.67 | 15.15 | 17.88 | 20.8 | 17.38 |
| 34 | WOMBAT | 19.26 | 20.72 | 21.13 | 22.02 | 20.78 | 5.33 | 7.35 | 9.38 | 12.73 | 8.698 |
| LSD at 5% | | 0.56 | | | | | 0.63 | | | | |
| LSD at 1% | | 0.73 | | | | | 0.83 | | | | |
|  |  |  |  |  |  |  |  |  |  |  |  |
|  |  |  |  |  |  |  |  |  |  |  |  |

Table S4. Mean performance of of panicle length and productive tillers of each growing season and over two growing seasons (mean±SD).

|  | Genotype Name | **Panicle length (cm)** | | | **No. of productive tillers/plant** | | |
| --- | --- | --- | --- | --- | --- | --- | --- |
| No. |  | **Y1** | **Y2** | **Y1+Y2** | **Y1** | **Y2** | **Y1+Y2** |
| 1 | Giza 171 | 12±10.7 | 13.6±11 | 12.8±10.9 | 10.7±9.9 | 12.5±10.2 | 11.6±10.1 |
| 2 | Giza 172 | 13.4±12 | 15±12.3 | 14.2±12.2 | 10.3±9.2 | 11.4±9.3 | 10.9±9.3 |
| 3 | Giza 176 | 12.7±11.5 | 14.4±11.8 | 13.6±11.7 | 9.7±8.6 | 11.6±9.6 | 10.7±9.1 |
| 4 | Giza 177 | 11.8±10.5 | 13.1±10.6 | 12.5±10.6 | 8.2±7.2 | 9.7±7.7 | 9±7.5 |
| 5 | Giza 178 | 12.1±10.7 | 13.4±10.9 | 12.8±10.8 | 9.3±8.6 | 12.2±10.2 | 10.8±9.4 |
| 6 | Giza 179 | 11.8±10.9 | 13.4±10.8 | 12.6±10.9 | 10±8.9 | 12±10.1 | 11±9.5 |
| 7 | Sakha 101 | 12.4±11.1 | 13.8±11.2 | 13.1±11.2 | 10.6±9.4 | 12.5±10 | 11.6±9.7 |
| 8 | Sakha 102 | 11.9±10.8 | 13.8±11 | 12.9±10.9 | 8.5±7.5 | 9.9±7.9 | 9.2±7.7 |
| 9 | Sakha 104 | 12.2±11 | 13.5±10.7 | 12.9±10.9 | 10±8.7 | 11.9±9.3 | 11±9 |
| 10 | Sakha 105 | 12.2±10.9 | 13.5±11.1 | 12.9±11 | 8.4±7.3 | 9.9±7.9 | 9.2±7.6 |
| 11 | Sakha 106 | 12±10.8 | 13.3±10.8 | 12.7±10.8 | 8.8±7.7 | 9.8±7.7 | 9.3±7.7 |
| 12 | Sakha 107 | 11.4±10.3 | 12.8±10.4 | 12.1±10.4 | 9.9±8.6 | 11.7±9.5 | 10.8±9.1 |
| 13 | Reiho | 11.3±10 | 12.9±10.3 | 12.1±10.2 | 10.3±9.2 | 11.8±9.6 | 11.1±9.4 |
| 14 | HR 20654-54-3-5 | 11.6±10.2 | 12.7±10.3 | 12.2±10.3 | 9.5±7.9 | 10.9±8.4 | 10.2±8.2 |
| 15 | IR 68333-R-R-B-19 | 12.4±11.3 | 13.6±11.4 | 13±11.4 | 10.8±9.6 | 12.4±10 | 11.6±9.8 |
| 16 | IR 11K 305A | 10.5±9.2 | 11.9±9.7 | 11.2±9.5 | 10.9±9.4 | 12.4±10.1 | 11.7±9.8 |
| 17 | IR 12K 269 | 10.8±9.9 | 12.6±10.1 | 11.7±10 | 11±9.5 | 12.6±10 | 11.8±9.8 |
| 18 | MILYANG 240 | 11.7±10.5 | 13±10.3 | 12.4±10.4 | 10.4±9.3 | 12.6±10.4 | 11.5±9.9 |
| 19 | Korea 14 | 11.3±10 | 12.7±9.9 | 12±10 | 10.4±9.4 | 11.9±9.8 | 11.2±9.6 |
| 20 | IR 83106-B-B-2 | 13.2±12.2 | 15±12.1 | 14.1±12.2 | 9.7±8.4 | 11.3±9.5 | 10.5±9 |
| 21 | SKC 2015-1 | 10.9±9.6 | 12±9.5 | 11.5±9.6 | 9.9±8.7 | 12.1±9.2 | 11±9 |
| 22 | SKC 2015-2 | 11.2±10.2 | 13.1±10.5 | 12.2±10.4 | 9.3±8.1 | 10.7±8.4 | 10±8.3 |
| 23 | GZ 9730-1-1-1-1 | 12.9±11.2 | 14.2±11.5 | 13.6±11.4 | 10.7±9.4 | 12.3±10.3 | 11.5±9.9 |
| 24 | GZ 9730-1-1-3-2 | 10.8±9.9 | 12.7±9.9 | 11.8±9.9 | 10.8±9.6 | 13±10.4 | 11.9±10 |
| 25 | GZ 9626-2-1-3-2 | 11.3±9.8 | 12.5±10 | 11.9±9.9 | 10.8±9.5 | 12.2±9.9 | 11.5±9.7 |
| 26 | GZ 6296-12-1-2-1 | 11.4±10.3 | 12.7±10.1 | 12.1±10.2 | 11.2±10 | 12.6±10.3 | 11.9±10.2 |
| 27 | IET 1444 | 12.6±11.6 | 13.7±11.1 | 13.2±11.4 | 10.6±9.6 | 12.8±10.4 | 11.7±10 |
| 28 | GZ 1368-S-5-4 | 11.9±10.7 | 13.1±10.5 | 12.5±10.6 | 10.6±9.5 | 12.6±10.3 | 11.6±9.9 |
| 29 | GZ 6903-1-2-2-1 | 11.1±9.9 | 12.6±10.1 | 11.9±10 | 9.7±8.5 | 11.6±9.4 | 10.7±9 |
| 30 | CIASEM | 13.1±11.5 | 14.3±11.6 | 13.7±11.6 | 11.2±10 | 13±10.7 | 12.1±10.4 |
| 31 | Sanakevelle | 13.5±12.3 | 15.5±12.8 | 14.5±12.6 | 6±4.8 | 7.4±5.7 | 6.7±5.3 |
| 32 | Carola | 12.8±11.4 | 14.3±11.4 | 13.6±11.4 | 7±6.4 | 9±7.4 | 8±6.9 |
| 33 | I Geo Tze | 11.5±10 | 13.1±10.5 | 12.3±10.3 | 9.9±8.4 | 12.1±9.7 | 11±9.1 |
| 34 | WOMBAT | 11.9±10.7 | 13.5±10.6 | 12.7±10.7 | 5.3±4.4 | 6.8±5.2 | 6.1±4.8 |

Table S5. Mean performance of spikelet fertility and harvest index traits over two growing seasons

| No. | Genotypes | **Fertility percentage (%)** | | | | | | **Harvest Index (%)** | | | | |
| --- | --- | --- | --- | --- | --- | --- | --- | --- | --- | --- | --- | --- |
|  |  | SD1 | SD2 | SD3 | SD4 | Mean | SD1 | | SD2 | SD3 | SD4 | Mean |
| 1 | Giza 171 | 77.51 | 82.62 | 90.53 | 93.14 | 85.95 | 30.57 | | 31.71 | 34.98 | 37.64 | 33.73 |
| 2 | Giza 172 | 84.16 | 85.33 | 91.85 | 94.54 | 88.97 | 31.4 | | 32.9 | 36.33 | 38.28 | 34.73 |
| 3 | Giza 176 | 82.36 | 84.18 | 91.33 | 92.91 | 87.7 | 31.77 | | 32.98 | 35.72 | 39.21 | 34.92 |
| 4 | Giza 177 | 82.19 | 83.73 | 92.87 | 95.44 | 88.56 | 32.98 | | 34.55 | 38.19 | 40.11 | 36.46 |
| 5 | Giza 178 | 74.61 | 79.97 | 84.59 | 90.58 | 82.44 | 29.38 | | 31.5 | 35.56 | 37.07 | 33.38 |
| 6 | Giza 179 | 81.98 | 82.47 | 86.26 | 92.08 | 85.7 | 31.42 | | 33.03 | 36.68 | 38.1 | 34.81 |
| 7 | Sakha 101 | 83.43 | 84.17 | 90.72 | 93.85 | 88.04 | 31.66 | | 33.62 | 36.22 | 41.16 | 35.67 |
| 8 | Sakha 102 | 83.03 | 84.69 | 92.63 | 95.54 | 88.97 | 29.29 | | 32.17 | 35.85 | 38.4 | 33.93 |
| 9 | Sakha 104 | 82.98 | 83.99 | 90.96 | 94.75 | 88.17 | 31.99 | | 33.39 | 36.85 | 39.38 | 35.4 |
| 10 | Sakha 105 | 75.92 | 83.04 | 92.81 | 95.51 | 86.82 | 31.45 | | 33.73 | 34.31 | 36.34 | 33.96 |
| 11 | Sakha 106 | 84.61 | 85.73 | 92.34 | 95.83 | 89.63 | 31.53 | | 34.25 | 36.07 | 38.61 | 35.12 |
| 12 | Sakha 107 | 83.48 | 84.73 | 92.43 | 93.2 | 88.46 | 31.14 | | 34.66 | 36.87 | 39.99 | 35.67 |
| 13 | Reiho | 84.59 | 85.51 | 92.81 | 96.55 | 89.87 | 31.23 | | 33.95 | 34.89 | 37.84 | 34.48 |
| 14 | HR 20654-54-3-5 | 83.49 | 84.66 | 92.48 | 94.7 | 88.83 | 34.51 | | 36.58 | 38.55 | 41.6 | 37.81 |
| 15 | IR 68333-R-R-B-19 | 82 | 82.37 | 90.18 | 93.95 | 87.13 | 31.93 | | 33.78 | 36.64 | 41.26 | 35.9 |
| 16 | IR 11K 305A | 74.92 | 77.05 | 89.93 | 92.45 | 83.59 | 30.76 | | 32.19 | 35.25 | 40.87 | 34.77 |
| 17 | IR 12K 269 | 80.82 | 81.37 | 91.23 | 93.6 | 86.76 | 28.94 | | 32.54 | 35.17 | 40.3 | 34.24 |
| 18 | MILYANG 240 | 80.86 | 82.64 | 84.6 | 92.22 | 85.08 | 32.8 | | 34.98 | 37.01 | 41.83 | 36.66 |
| 19 | Korea 14 | 81 | 81.92 | 90.94 | 93.87 | 86.93 | 32.41 | | 36.02 | 38.58 | 40.53 | 36.89 |
| 20 | IR 83106-B-B-2 | 74.74 | 79.66 | 89.47 | 91.41 | 83.82 | 29.82 | | 31.49 | 35.83 | 39.32 | 34.12 |
| 21 | SKC 2015-1 | 76.08 | 81.58 | 91.68 | 93.64 | 85.75 | 31.9 | | 34.09 | 38 | 39.15 | 35.79 |
| 22 | SKC 2015-2 | 81.6 | 83.08 | 89.92 | 92.76 | 86.84 | 28.97 | | 32.13 | 33.78 | 37.29 | 33.04 |
| 23 | GZ 9730-1-1-1-1 | 82.7 | 84.57 | 92.57 | 94.24 | 88.52 | 32.87 | | 33.36 | 38.54 | 39.81 | 36.15 |
| 24 | GZ 9730-1-1-3-2 | 82.71 | 83.9 | 89.44 | 95.21 | 87.82 | 32.12 | | 35.03 | 36.27 | 39.88 | 35.83 |
| 25 | GZ 9626-2-1-3-2 | 83.15 | 84.41 | 92.32 | 94.91 | 88.7 | 34.04 | | 35.53 | 37.54 | 39.17 | 36.57 |
| 26 | GZ 6296-12-1-2-1 | 81.78 | 82.54 | 91.5 | 92.63 | 87.11 | 30.32 | | 33.48 | 36.51 | 39.31 | 34.91 |
| 27 | IET 1444 | 79.64 | 81.18 | 90.1 | 92.16 | 85.77 | 31.46 | | 32.85 | 36.25 | 40.19 | 35.19 |
| 28 | GZ 1368-S-5-4 | 81.46 | 82.67 | 90.72 | 91.47 | 86.58 | 28.87 | | 30.57 | 35.38 | 40.34 | 33.79 |
| 29 | GZ 6903-1-2-2-1 | 82.39 | 83.41 | 92.68 | 93.86 | 88.09 | 33.62 | | 34.69 | 37.9 | 42.38 | 37.15 |
| 30 | CIASEM | 81.73 | 81.08 | 86.67 | 90.23 | 84.93 | 25.19 | | 30.91 | 33.84 | 38.05 | 32 |
| 31 | Sanakevelle | 82.31 | 83.92 | 92.49 | 94.06 | 88.2 | 28.18 | | 32.01 | 34.8 | 37.17 | 33.04 |
| 32 | Carola | 80.6 | 83.19 | 91.06 | 91.92 | 86.69 | 29.22 | | 32.33 | 33.79 | 35.11 | 32.61 |
| 33 | I Geo Tze | 75.51 | 80.52 | 90.97 | 91.65 | 84.66 | 28.47 | | 31.82 | 35.53 | 41.85 | 34.42 |
| 34 | WOMBAT | 82.85 | 83.84 | 93.89 | 94.44 | 88.76 | 28.27 | | 31.33 | 35.09 | 40.92 | 33.9 |
| LSD at 5% | | 0.85 | | | | | | 0.57 | | | | |
| LSD at 1% | | 1.11 | | | | | | 0.75 | | | | |

Table S6. Mean performance of spikelet fertility and harvest index traits of each growing season and over two growing seasons (mean±SD).

|  |  | **Fertility %** | | | **Harvest Index (%)** | | |
| --- | --- | --- | --- | --- | --- | --- | --- |
| No. |  | **Y1** | **Y2** | **Y1+Y2** | **Y1** | **Y2** | **Y1+Y2** |
| 1 | Giza 171 | 46.5±43.7 | 52.3±44.4 | 49.4±44.1 | 19±17.6 | 21.8±17.7 | 20.4±17.7 |
| 2 | Giza 172 | 47.1±44.5 | 53.3±44.4 | 50.2±44.5 | 19.7±18.2 | 22.1±18.2 | 20.9±18.2 |
| 3 | Giza 176 | 46.6±43.7 | 52.7±44.7 | 49.7±44.2 | 19.8±17.8 | 22.8±19 | 21.3±18.4 |
| 4 | Giza 177 | 47.6±44.4 | 54.3±45.1 | 51±44.8 | 20.9±19.2 | 23.7±19.3 | 22.3±19.3 |
| 5 | Giza 178 | 43.9±41 | 50.3±42.8 | 47.1±41.9 | 19.5±17.5 | 21.5±17.8 | 20.5±17.7 |
| 6 | Giza 179 | 44.8±41.9 | 51.4±43.6 | 48.1±42.8 | 19.8±18.1 | 22.3±18.5 | 21.1±18.3 |
| 7 | Sakha 101 | 46.5±44.1 | 52.5±44.7 | 49.5±44.4 | 20.4±18.5 | 23.4±19.5 | 21.9±19 |
| 8 | Sakha 102 | 47.1±44.7 | 53.7±45.7 | 50.4±45.2 | 19.6±17.6 | 22.5±18.4 | 21.1±18 |
| 9 | Sakha 104 | 47±43.4 | 53.1±44.8 | 50.1±44.1 | 20±18.3 | 23.2±19.2 | 21.6±18.8 |
| 10 | Sakha 105 | 47.2±44.1 | 53.9±45.6 | 50.6±44.9 | 19.2±17.2 | 21.4±17.4 | 20.3±17.3 |
| 11 | Sakha 106 | 47.7±44.7 | 53.6±45.4 | 50.7±45.1 | 20±18.3 | 22.7±19 | 21.4±18.7 |
| 12 | Sakha 107 | 47±44.2 | 52.6±44.4 | 49.8±44.3 | 20.3±18.5 | 23.3±19.4 | 21.8±19 |
| 13 | Reiho | 47.6±45 | 54.1±46 | 50.9±45.5 | 19.5±17.5 | 22.1±18 | 20.8±17.8 |
| 14 | HR 20654-54-3-5 | 47.2±44.5 | 53.7±45.5 | 50.5±45 | 21.3±19.5 | 24.6±20.2 | 23±19.9 |
| 15 | IR 68333-R-R-B-19 | 46.2±43.1 | 52.2±44.5 | 49.2±43.8 | 20.2±18.5 | 24±19.7 | 22.1±19.1 |
| 16 | IR 11K 305A | 44.7±42.3 | 51.4±43.8 | 48.1±43.1 | 19.3±17.8 | 23±19 | 21.2±18.4 |
| 17 | IR 12K 269 | 46.1±43.5 | 52.8±44.7 | 49.5±44.1 | 19.7±17.9 | 22.9±18.6 | 21.3±18.3 |
| 18 | MILYANG 240 | 44.2±41.7 | 51.1±42.6 | 47.7±42.2 | 20.4±19.1 | 24.3±19.9 | 22.4±19.5 |
| 19 | Korea 14 | 46.1±43.6 | 52.8±44.6 | 49.5±44.1 | 21.2±19.5 | 24.2±19.8 | 22.7±19.7 |
| 20 | IR 83106-B-B-2 | 45.2±42.9 | 51.2±43.4 | 48.2±43.2 | 19.5±17.6 | 22.5±18.6 | 21±18.1 |
| 21 | SKC 2015-1 | 46.5±43.4 | 52.7±44.9 | 49.6±44.2 | 20.6±18.7 | 23.4±18.9 | 22±18.8 |
| 22 | SKC 2015-2 | 45.6±43.7 | 52.4±44.3 | 49±44 | 18.7±17.3 | 21.7±17.8 | 20.2±17.6 |
| 23 | GZ 9730-1-1-1-1 | 47.2±43.9 | 53.6±45.2 | 50.4±44.6 | 20.5±18.9 | 23.8±19.5 | 22.2±19.2 |
| 24 | GZ 9730-1-1-3-2 | 46.5±43.4 | 52.7±44.8 | 49.6±44.1 | 20.4±18.7 | 23±18.8 | 21.7±18.8 |
| 25 | GZ 9626-2-1-3-2 | 47.1±44.1 | 53.6±45.3 | 50.4±44.7 | 21.1±19 | 23.1±18.9 | 22.1±19 |
| 26 | GZ 6296-12-1-2-1 | 46.2±43.7 | 52.1±44.2 | 49.2±44 | 20.4±18.3 | 22.9±18.9 | 21.7±18.6 |
| 27 | IET 1444 | 45.7±42.9 | 52.1±44.2 | 48.9±43.6 | 20.2±18 | 22.9±18.7 | 21.6±18.4 |
| 28 | GZ 1368-S-5-4 | 46.1±43.3 | 51.6±43.8 | 48.9±43.6 | 19.3±17.6 | 22.6±18.8 | 21±18.2 |
| 29 | GZ 6903-1-2-2-1 | 47.5±44.1 | 53.8±45.1 | 50.7±44.6 | 20.9±19 | 24.6±20.2 | 22.8±19.6 |
| 30 | CIASEM | 44.9±41.7 | 50.7±42.9 | 47.8±42.3 | 19±17.3 | 21.8±18 | 20.4±17.7 |
| 31 | Sanakevelle | 47.2±44.5 | 53.1±44.7 | 50.2±44.6 | 19.3±17.5 | 22.2±18 | 20.8±17.8 |
| 32 | Carola | 46.4±43.2 | 52.3±44.4 | 49.4±43.8 | 18.6±16.8 | 21±16.9 | 19.8±16.9 |
| 33 | I Geo Tze | 46±42.9 | 51.7±43.9 | 48.9±43.4 | 19.8±18 | 23.5±19.5 | 21.7±18.8 |
| 34 | WOMBAT | 47.6±44.3 | 53.9±45.7 | 50.8±45 | 19.6±17.8 | 23.2±18.9 | 21.4±18.4 |

Table S7. Mean performance of grain yield and biomass traits over two growing seasons

| No. | Genotypes | **Grain Yield/plant (g)** | | | | | **Biomass (g)** | | | | |
| --- | --- | --- | --- | --- | --- | --- | --- | --- | --- | --- | --- |
|  |  | SD1 | SD2 | SD3 | SD4 | Mean | SD1 | SD2 | SD3 | SD4 | Mean |
| 1 | Giza 171 | 20.85 | 27.78 | 31.28 | 35.06 | 28.74 | 71.58 | 83.34 | 91.99 | 109.3 | 89.06 |
| 2 | Giza 172 | 21.6 | 24.94 | 33.17 | 36.47 | 29.05 | 73.92 | 80.31 | 94.22 | 112.2 | 90.16 |
| 3 | Giza 176 | 25.72 | 20.57 | 33.61 | 38.2 | 29.53 | 77.7 | 82.48 | 94.13 | 102.1 | 89.11 |
| 4 | Giza 177 | 23.07 | 28.15 | 33.16 | 37.89 | 30.57 | 74.32 | 81.91 | 88.08 | 94.53 | 84.71 |
| 5 | Giza 178 | 21.06 | 27.34 | 31.91 | 40.71 | 30.26 | 71.83 | 78.44 | 89.81 | 101.5 | 85.41 |
| 6 | Giza 179 | 21.65 | 27.18 | 34.56 | 42.36 | 31.44 | 72.1 | 82.31 | 93.62 | 105.1 | 88.28 |
| 7 | Sakha 101 | 23.64 | 30.93 | 35.34 | 42.17 | 33.02 | 74.78 | 87.67 | 97.63 | 102.5 | 90.63 |
| 8 | Sakha 102 | 23.6 | 28.62 | 31.88 | 37.89 | 30.5 | 80.62 | 89.07 | 94.18 | 104.4 | 92.07 |
| 9 | Sakha 104 | 27.4 | 31.21 | 35.77 | 41.73 | 34.03 | 85.7 | 92.75 | 98.47 | 106 | 95.73 |
| 10 | Sakha 105 | 22.35 | 25.96 | 31.87 | 37.6 | 29.45 | 71.12 | 80.53 | 89.63 | 99.54 | 85.21 |
| 11 | Sakha 106 | 23.39 | 29.86 | 32.4 | 38.27 | 30.98 | 78.07 | 86.97 | 93.17 | 99.13 | 89.34 |
| 12 | Sakha 107 | 25.27 | 30.67 | 33.81 | 38.32 | 32.02 | 81.26 | 86.73 | 91.77 | 95.86 | 88.91 |
| 13 | Reiho | 20.4 | 25.17 | 29.62 | 35.56 | 27.69 | 65.38 | 74.26 | 85.03 | 94.03 | 79.68 |
| 14 | HR 20654-54-3-5 | 22.67 | 22.82 | 29.7 | 36.16 | 27.84 | 65.73 | 69.62 | 77.1 | 86.96 | 74.85 |
| 15 | IR 68333-R-R-B-19 | 18.38 | 22.11 | 33.09 | 39.28 | 28.22 | 57.67 | 67.14 | 88.8 | 95.27 | 77.22 |
| 16 | IR 11K 305A | 21.03 | 24.44 | 28.28 | 37.86 | 27.9 | 68.48 | 75.98 | 80.32 | 95.34 | 80.03 |
| 17 | IR 12K 269 | 21.24 | 24.97 | 29.87 | 39.74 | 28.96 | 73.47 | 78.7 | 85.05 | 98.63 | 83.96 |
| 18 | MILYANG 240 | 16.61 | 21.55 | 28.89 | 37.2 | 26.06 | 50.72 | 61.62 | 78.19 | 88.96 | 69.87 |
| 19 | Korea 14 | 21.32 | 23.79 | 29.35 | 33.73 | 27.05 | 65.8 | 75.45 | 81.29 | 87.61 | 77.54 |
| 20 | IR 83106-B-B-2 | 20.32 | 21.74 | 29.09 | 36.21 | 26.84 | 68.17 | 75.14 | 81.28 | 92.2 | 79.2 |
| 21 | SKC 2015-1 | 21.45 | 23.08 | 23.93 | 32.71 | 25.29 | 67.31 | 75.98 | 84.63 | 89.08 | 79.25 |
| 22 | SKC 2015-2 | 20.4 | 23.11 | 24.24 | 34.74 | 25.62 | 70.45 | 76.74 | 81.21 | 93.26 | 80.42 |
| 23 | GZ 9730-1-1-1-1 | 21.39 | 26.88 | 31.14 | 41.61 | 30.26 | 76.51 | 82.73 | 88.23 | 104.7 | 88.04 |
| 24 | GZ 9730-1-1-3-2 | 20.59 | 26.29 | 28.17 | 38.68 | 28.43 | 71.24 | 78.77 | 85.55 | 97.07 | 83.16 |
| 25 | GZ 9626-2-1-3-2 | 19.66 | 23.04 | 26.39 | 37.84 | 26.73 | 69.62 | 77.92 | 83.68 | 96.69 | 81.98 |
| 26 | GZ 6296-12-1-2-1 | 20.84 | 24.2 | 28.11 | 39.87 | 28.26 | 68.75 | 76.55 | 84.23 | 101.5 | 82.76 |
| 27 | IET 1444 | 21.81 | 26.55 | 31.91 | 38.86 | 29.78 | 69.43 | 80.95 | 88.19 | 96.77 | 83.84 |
| 28 | GZ 1368-S-5-4 | 21.01 | 25.91 | 33.47 | 37.84 | 29.56 | 72.91 | 80.84 | 91.15 | 94.5 | 84.85 |
| 29 | GZ 6903-1-2-2-1 | 16.89 | 24.28 | 32.14 | 41.04 | 28.59 | 74.89 | 81.61 | 88.56 | 96.87 | 85.48 |
| 30 | CIASEM | 11.99 | 14.88 | 18.52 | 23.43 | 17.21 | 57.08 | 64.87 | 72.63 | 80.97 | 68.89 |
| 31 | Sanakevelle | 18.85 | 21.79 | 24.34 | 28.85 | 23.46 | 66.96 | 71.12 | 76.01 | 83.05 | 74.29 |
| 32 | Carola | 14.81 | 16.36 | 18.98 | 25.82 | 18.99 | 62.45 | 69.89 | 74.59 | 81.14 | 72.02 |
| 33 | I Geo Tze | 19.31 | 21.83 | 25.71 | 33.07 | 24.98 | 65.69 | 70.85 | 72.44 | 82.29 | 72.82 |
| 34 | WOMBAT | 15.49 | 20.95 | 30.76 | 34.86 | 25.52 | 60 | 70.36 | 82.53 | 89.13 | 75.51 |
| LSD at 5% | | 0.48 | | | | | 0.93 | | | | |
| LSD at 1% | | 0.64 | | | | | 1.22 | | | | |

Table S8. Mean performance of grain yield and biomass traits of each growing season and over two growing seasons (mean±SD)

|  |  | **Grain Yield/plant (g)** | | | **Biomass (g)** | | |
| --- | --- | --- | --- | --- | --- | --- | --- |
| No. |  | **Y1** | **Y2** | **Y1+Y2** | **Y1** | **Y2** | **Y1+Y2** |
| 1 | Giza 171 | 22.9±14.9 | 25.4±12.6 | 24.2±13.8 | 50.1±47.7 | 60.1±51.2 | 55.1±49.5 |
| 2 | Giza 172 | 23.7±15.6 | 25.6±13.1 | 24.7±14.4 | 51±47.7 | 60.7±51.4 | 55.9±49.6 |
| 3 | Giza 176 | 24.3±16 | 25±14 | 24.7±15 | 50.3±45.6 | 59.3±48.8 | 54.8±47.2 |
| 4 | Giza 177 | 24.2±15.6 | 27.2±13.7 | 25.7±14.7 | 48.1±43.9 | 54.3±44.6 | 51.2±44.3 |
| 5 | Giza 178 | 24.5±16.5 | 27.3±14.1 | 25.9±15.3 | 48±44.2 | 57.5±47.5 | 52.8±45.9 |
| 6 | Giza 179 | 25.7±17.5 | 28.2±14.9 | 27±16.2 | 50±46.3 | 58.8±49.2 | 54.4±47.8 |
| 7 | Sakha 101 | 26.4±17.3 | 29.8±15 | 28.1±16.2 | 52.2±48 | 60.1±49.3 | 56.2±48.7 |
| 8 | Sakha 102 | 24.1±15.6 | 26.9±13.5 | 25.5±14.6 | 52.1±47.8 | 60.7±49.8 | 56.4±48.8 |
| 9 | Sakha 104 | 26.8±17.1 | 29.8±14.8 | 28.3±16 | 54.3±49.3 | 60.8±50.5 | 57.6±49.9 |
| 10 | Sakha 105 | 23.8±15.6 | 25.9±13.2 | 24.9±14.4 | 48.6±44.6 | 56±47.2 | 52.3±45.9 |
| 11 | Sakha 106 | 24.4±15.6 | 27.5±13.7 | 26±14.7 | 50.9±46.1 | 56.9±47.8 | 53.9±47 |
| 12 | Sakha 107 | 24.7±15.8 | 28.4±14.2 | 26.6±15 | 50.7±46.2 | 56±46.2 | 53.4±46.2 |
| 13 | Reiho | 22.2±14.5 | 24.8±12.6 | 23.5±13.6 | 46.4±42.2 | 53.4±43.2 | 49.9±42.7 |
| 14 | HR 20654-54-3-5 | 22±14.6 | 24.5±12.9 | 23.3±13.8 | 42.3±38.9 | 48.4±40.8 | 45.4±39.9 |
| 15 | IR 68333-R-R-B-19 | 23.6±16 | 26.1±14.2 | 24.9±15.1 | 46±42.3 | 53.1±44.7 | 49.6±43.5 |
| 16 | IR 11K 305A | 22.4±15.1 | 24.8±13 | 23.6±14.1 | 45.3±41.4 | 53.2±43.4 | 49.3±42.4 |
| 17 | IR 12K 269 | 23.4±15.9 | 26±13.8 | 24.7±14.9 | 46.8±43.5 | 55.4±45.5 | 51.1±44.5 |
| 18 | MILYANG 240 | 21.5±15 | 24.2±13 | 22.9±14 | 41.6±39 | 48.5±39.3 | 45.1±39.2 |
| 19 | Korea 14 | 21.6±14.1 | 23.7±11.9 | 22.7±13 | 44.4±40.4 | 50.2±42.1 | 47.3±41.3 |
| 20 | IR 83106-B-B-2 | 21.6±14.5 | 24.1±12.9 | 22.9±13.7 | 44.7±40.3 | 52.1±43.2 | 48.4±41.8 |
| 21 | SKC 2015-1 | 19.3±12.9 | 22.2±11.5 | 20.8±12.2 | 45.5±41.3 | 51.7±43.2 | 48.6±42.3 |
| 22 | SKC 2015-2 | 20.2±13.5 | 22.7±12 | 21.5±12.8 | 44.8±40.3 | 53.1±44.3 | 49±42.3 |
| 23 | GZ 9730-1-1-1-1 | 24.1±16.4 | 27.6±14.6 | 25.9±15.5 | 48.5±45.3 | 58.1±48.9 | 53.3±47.1 |
| 24 | GZ 9730-1-1-3-2 | 22.2±15.2 | 25.9±13.6 | 24.1±14.4 | 46.5±42.7 | 56.1±46 | 51.3±44.4 |
| 25 | GZ 9626-2-1-3-2 | 21.4±14.5 | 24.2±13.1 | 22.8±13.8 | 46.3±42.5 | 54.1±45.1 | 50.2±43.8 |
| 26 | GZ 6296-12-1-2-1 | 22.8±15.5 | 25.4±13.8 | 24.1±14.7 | 46.9±42.4 | 55.7±47.2 | 51.3±44.8 |
| 27 | IET 1444 | 23.8±15.7 | 26.8±13.7 | 25.3±14.7 | 49.9±42.7 | 57.1±46.3 | 53.5±44.5 |
| 28 | GZ 1368-S-5-4 | 23.8±15.8 | 26.7±13.7 | 25.3±14.8 | 49±44.4 | 54.9±46 | 52±45.2 |
| 29 | GZ 6903-1-2-2-1 | 23.6±16.4 | 27.1±14.6 | 25.4±15.5 | 48±44.7 | 55.7±46.4 | 51.9±45.6 |
| 30 | CIASEM | 14.2±9.5 | 15.5±8 | 14.9±8.8 | 39.6±35.8 | 46.3±37.9 | 43±36.9 |
| 31 | Sanakevelle | 18±11.6 | 21±10.4 | 19.5±11 | 41.8±37.2 | 47.4±38.6 | 44.6±37.9 |
| 32 | Carola | 15±9.7 | 17.2±9.1 | 16.1±9.4 | 41.7±37 | 47.5±38.9 | 44.6±38 |
| 33 | I Geo Tze | 19.8±13.2 | 22.2±11.5 | 21±12.4 | 41.2±36.7 | 47.7±38.4 | 44.5±37.6 |
| 34 | WOMBAT | 21.2±14.6 | 23.8±12.8 | 22.5±13.7 | 43.6±40 | 50.8±42 | 47.2±41 |
